# Supplementary material for: Phosphatase SHP2 pathogenic mutations enhance activity by altering conformational sampling
Source: Proc Natl Acad Sci U S A. 2026 Jan 13;123(3):e2513851123. doi: 10.1073/pnas.2513851123 (PMC12818432; doi:10.1073/pnas.2513851123)
Supplement: Supplementary file 1 — Appendix 01 (PDF) [file pnas.2513851123.sapp.pdf]

Supporting Information for

Phosphatase SHP2 pathogenic mutations enhance activity by altering conformational sampling

Andrew W. Glaser[1]†, Ricardo A. P. Pádua[1,2]†, Adedolapo M. Ojoawo[1,2]†, Camille Sullivan[1], Dorothee Kern[1,2]

[1]Department of Biochemistry and Biophysics, Brandeis University and Howard Hughes Medical Institute, Waltham, MA, USA
[2]Department of Integrative Structural and Computational Biology, Scripps Research Institute and Howard Hughes Medical Institute, La Jolla, CA, USA
† These authors contributed equally.

Corresponding author: dkern@scripps.edu

This file includes:

Materials and Methods
Figs. S1 to S20
Tables S1 to S2
SI References

Materials and Methods

Preparation of Recombinant Proteins

The optimized DNA sequence encoding the human wild-type SHP2 (FL-WT) lacking the C-terminal tail (residues 1–529, UniProt: Q06124-2) was synthesized with an N-terminal Tobacco Etch Virus protease (TEV) cleavage site and sub-cloned into pET-28a(+) expression vector containing a His-tag on the N-terminus by Genscript, as described previously.(1) The N-SH2 (residues 1–106) and C-SH2 (residues 104-217) expression plasmids were generated as described previously.(1) A codon optimized gene of the N-SH2 residues 4-103 (N-SH2$^{WT\text{-}short}$) preceded by the sequence ENLYFQGSHM was synthesized and cloned into pET-14b between *Nde*I and BamHI restriction sites (Genscript) to reproduce the experiments by Lee et al.(2) Point mutations of SHP2 constructs (T42S, T42A, and E139D) were introduced using the QuikChange Lightning mutagenesis kit (Agilent Technologies) with primers synthesized by Azenta. Mutagenesis was confirmed through plasmid Sanger sequencing (Azenta). All fusion proteins were expressed in *Escherichia coli* BL21(DE3) competent cells (NEB). Full-length proteins for use in enzymatic assays were expressed in *E. coli* grown in Terrific broth (Thermo Fisher Scientific). Isolated SH2 domains uniformly labeled with $^{15}N/^{13}C$ were expressed in *E. coli* grown in M9 minimal medium prepared with H_2O. The medium was supplemented with 1.0 g/L of $^{15}NH_4Cl$ and 2.0 g/L of U-[^{13}C]-glucose (Cambridge Isotope Laboratories). Full-length proteins uniformly labeled with $^{15}N/^{13}C/^{2}H$ were expressed in *E. coli* grown in M9 minimal medium prepared with 99.8% D_2O (Cambridge Isotope Laboratories). The medium contained 1.0 g/L of $^{15}NH_4Cl$ and 2.0 g/L of U-[^{2}H, ^{13}C]-glucose as the sole carbon source (Cambridge Isotope Laboratories). Cells were then lysed by sonication (20 s on and 20 s off at 33 watt at 4 °C for 20-30 minutes) and proteins were purified through affinity chromatography on a HisTrap column (GE Healthcare). The His-tag was subsequently cleaved off with in-house made His-tagged TEV protease overnight at 4 °C while dialyzing against HisTrap binding buffer (50 mM Tris pH 8, 500 mM NaCl, and 1 mM tris(2-carboxyethyl) phosphine (TCEP)). The cleaved protein was separated from His-tag and TEV protease by a second HisTrap purification. The flow through was collected and further purified by size exclusion chromatography on a HiLoad 16/600 Superdex 75 pg column (GE Healthcare) in storage buffer (50 mM Bis-Tris pH 6.5, 50 mM NaCl, 1 mM TCEP). All recombinant SHP2 constructs except for PTP contained three extraneous amino acids (GSG) preceding their natural sequences.

Preparation of Bis-Phosphorylated BTLA (2P-BTLA)

The DNA sequence for B- and T-lymphocyte attenuator (BTLA) residues 254-288 (Uniprot: Q7Z6A9) was synthesized and cloned into a pET-28b vector by Genscript, containing an N-terminal 6xHis-GB1 solubility tag followed by a TEV cleavage site. These constructs were transformed into *E. coli* BL21(DE3) cells, which were grown at 37 °C to an OD_{600} between 0.8 and 1.2. Protein expression was induced with 1 mM Isopropyl β-D-1-thiogalactopyranoside (IPTG) for 4 hours at 37 °C. After induction, cells were harvested by centrifugation and stored at -20 °C until purification.

Cell pellets were resuspended in a binding buffer containing 50 mM Tris, pH 8.0, and 6 M guanidine hydrochloride (GuHCl). The lysate was sonicated for 20-30 minutes (20 s on and 20 s off at 33 watt) at 4 °C, followed by centrifugation at 20,000 x g for 1 hour using a Beckman JA20 rotor. The soluble, denatured protein lysate was filtered through Whatman Puradisc 25mm PES sterile syringe filters and passed over Takara Bio Talon metal affinity resin (10 mL beads/L of cells) using the batch protocol. Unbound proteins were washed with 1 column volume (CV) of binding buffer plus 4 mM imidazole and 1 mM TCEP.

After washing, the protein-bound beads were rapidly diluted with refolding buffer containing 50 mM Tris, pH 8, 500 mM NaCl, 1 mM TCEP, 4 mM imidazole, and 0.1% Triton X-100, ensuring the final

concentration of GuHCl did not exceed 0.6 M. The detergent was removed from the Talon resin with buffer containing 50 mM Tris, pH 8, 500 mM NaCl, 4 mM imidazole, and 5 mM β-cyclodextrin before elution. The refolded protein was eluted with 50 mM Tris, pH 8, 500 mM NaCl, 250 mM imidazole, and 1 mM TCEP, then concentrated. The solubility tag was cleaved with TEV protease (1:50 ratio of TEV to peptide) overnight at room temperature in dialysis buffer without imidazole (50 mM Tris, pH 8, 500 mM NaCl, and 1 mM TCEP). Most of the protein refolded successfully and was efficiently cleaved, resulting in the successful purification of unphosphorylated activator peptides. A final HisTrap purification was performed to remove TEV protease, yielding nearly pure activator peptides.

Activator peptide was mixed with 20 µM of the recombinantly expressed Src kinase domain, 5 mM ATP, and 50 mM $MgCl_2$ in 50 mM Tris pH 8.0, 500 mM NaCl, and 1 mM TCEP. The peptides were completely phosphorylated after one hour at 25 °C, as determined by ^{1}H NMR. The bis-phosphorylated BTLA peptide (2P-BTLA) was acidified with a 1:1 mixture of 1 M citric acid and 0.2% trifluoroacetic acid (TFA) in Milli-Q ultra-pure water.

The samples were then filtered through a 0.2 µm 13 mm PTFE syringe filter (Fisherbrand), and purified by high-performance liquid chromatography (HPLC) using a 0.1% TFA and acetonitrile gradient over a C18 ACE column. The phosphorylated peptides were dried using a SpeedVac vacuum concentrator, resuspended in 50 mM Bis-Tris, pH 6.5, 50 mM NaCl, and 1 mM TCEP, and stored at -80 °C until use. Phosphorylated peptide concentrations were determined by measuring absorbance at 205 nm.(3)

Steady-state Kinetics

Phosphatase activity was measured by monitoring the dephosphorylation of the synthetic substrate DiFMUP (6,8-difluoro-4-methy-lumbelliferyl phosphate) to the product DiFMU (Thermo Fisher Scientific). The reaction was carried out in activity buffer (50 mM Bis-Tris pH 6.5, 50 mM NaCl, 1 mM TCEP, 0.05% Tween 20, 0.3 mg/mL bovine serum albumin) at 25°C and was started by adding 10 µL of enzyme to 90 µL of substrate in a 96-well plate (Corning—Ref 3994). The absorbance at 366 nm was continuously monitored in a SpectraMax i3x plate reader (Molecular Devices) and converted to product concentration using a calibration curve obtained from a serial dilution of the pure product. Final enzyme concentrations used were 0.2–10 nM for FL proteins. Uncertainties were calculated from the standard deviation of the observations (n = 3).

Activation Assay

Phosphatase activity was measured in the presence of various concentrations of the activator 2P-BTLA in activity buffer (50 mM Bis-Tris pH 6.5, 50 mM NaCl, 1 mM TCEP, 0.05% Tween 20, 0.3 mg/mL bovine serum albumin) at 25°C. The assay was initiated by adding 10 µL of enzyme (for a final concentration of 0.2 nM) to 90 uL of solution containing substrate and varying concentrations of 2P-BTLA, with a final substrate concentration of 500 µM DiFMUP. The absorbance at 366 nm was converted into observed rates, which were plotted against the logarithmic concentration of 2P-BTLA and the K_{Act} values and uncertainties were obtained after fitting the AC_{50} function to the data.

Isothermal Titration Calorimetry

Genscript synthesized the mono-phosphorylated peptide motifs 1P-$GAB1_{N\text{-}term}$ (QVEpYLDLDLD), 1P-$BTLA_{N\text{-}term}$ (GIVpYASLNHS), 1P-$BTLA_{C\text{-}term}$ (PTEpYASICVR), and the unphosphorylated $GAB1_{N\text{-}term}$ peptide (QVEYLDLDLD). Protein samples were dialyzed against storage buffer and lyophilized phosphopeptides were dissolved in the same buffer. Concentrations of protein and peptide are displayed in **Fig. S3** and **Fig.**

S5. ITC experiments were carried out in a Nano ITC instrument (TA Instruments) at 25 °C. The titrations were performed by injecting 1.0 – 1.5 µL aliquots of phosphopeptide into the calorimeter cell containing a 170 µL solution of SH2 domain with a constant stirring speed of 250 rpm. The data were analyzed with the NanoAnalyze using the independent fit model. All the uncertainties were estimated by the native Statistics module with 1000 synthetic trials and 95% confidence level.

NMR Spectroscopy

All NMR data were collected at 298 K on Bruker AVANCE NEO/III or Varian VNMRS DD spectrometers ranging from 600 MHz to 900 MHz, equipped with cryogenic triple resonance probes. All 3D experiments performed for resonance assignments were collected with non-uniform sampling with a sampling rate of ~30%. All NMR datasets were processed using NMRPipe(4), and NUS data were reconstructed with SMILE(5) package. All backbone dynamics (R_1, R_2, hetNOE, ^1H-^{15}N CEST, ^1H-^{15}N CPMG) data were collected on a 600MHz spectrometer equipped with a cryogenic probe.

Phosphorylated Peptide Titration. NMR titration experiments were performed in a 50 mM Bis-Tris, pH 6.5, 50 mM NaCl, 1 mM TCEP, 1P-BTLA$_{N\text{-}term}$ was resuspended in the same buffer. Each N-SH2 variant (N-SH2^{WT}, N-SH2^{T42S}, N-SH2^{T42A}) was prepared at a concentration of 100 µM and ^1H-^{15}N TROSY-HSQC spectra were recorded across a series of titration points, with 1P-BTLA$_{N\text{-}term}$ concentrations ranging from 0.25 to 4 molar equivalents relative to protein.

Chemical Shift Perturbation (CSP) Analysis. CSPs values were derived using the equation:

$$\Delta\delta\ (ppm) = \sqrt{(\Delta\delta_H)^2 + (0.15 * \Delta\delta_N)^2}$$

where $\Delta\delta_H$ and $\Delta\delta_N$ represents changes in proton and nitrogen chemical shifts, respectively.

Determination of Fraction Bound. Residues with CSP values exceeding a threshold of 0.5 ppm were included in the fraction bound analysis. The fraction bound protein at each titration point was calculated from NMR peak volumes using the equation:

$$Fraction\ Bound = \frac{Volume_{Bound}}{Volume_{Apo} + Volume_{Bound}}$$

Binding Curve Simulation. Theoretical binding curves were simulated using the Langmuir binding model:

$$Fraction\ Bound = \frac{[L]}{K_D + [L]}$$

Where [L] is the ligand concentration and K_D is the dissociation constant for each variant. Experimental K_D values were as follows: 500 nM (N-SH2^{WT}), 120 nM (N-SH2^{T42S}), and 50 nM (N-SH2^{T42A}) as determined by ITC. The protein concentration was set to 100 µM, and the simulated fraction bound was calculated for each molar ratio of ligand to protein.

Unphosphorylated Peptide Titration. NMR titration experiments were conducted in a buffer containing 50 mM Bis-Tris, pH 6.5, 50 mM NaCl, and 1 mM TCEP. N-SH2^{WT} was prepared at a concentration of 100 µM, and ^1H-^{15}N TROSY-HSQC spectra were recorded across a series of titration points. Unphosphorylated GAB1$_{N\text{-}term}$ peptide (resuspended in the same buffer) concentrations ranged from 0.25 to 4 molar equivalents relative to protein. Data were analyzed by fitting CSPs as a function of ligand concentration to extract dissociation constants, using the equation:

$$\Delta\delta = \Delta\delta_{max} * \frac{[P]_0 + [L]_0 + K_D - \sqrt{([P]_0 + [L]_0 + K_D)^2 - 4[P]_0[L]_0}}{2P_0}$$

Where $[P]_0$ and $[L]_0$ are the total concentrations of protein and ligand, respectively, $\Delta\delta_{max}$ is the maximum CSP and K_D is the dissociation constant.

Phosphotyrosine titration. The N-SH2 variants (N-SH2^{WT}, N-SH2^{T42S}, N-SH2^{T42A}) were prepared at 100 µM in the same buffer conditions (50 mM Bis-Tris, pH 6.5, 50 mM NaCl, and 1 mM TCEP). Increasing concentrations of phosphotyrosine (resuspended in the same buffer) were titrated into the samples, ranging from 0.25 to 10 molar equivalents relative to protein. ^{1}H-^{15}N TROSY-HSQC spectra were recorded at each titration point, and dissociation constants were extracted by analyzing chemical shift perturbations, using the quadratic equation described above.

Backbone assignments. Backbone ^{1}H, ^{15}N, ^{13}C assignments were obtained on ~1.2 mM uniformly ^{15}N, ^{13}C labeled apo/bound form of N-SH2^{WT} and NSH2^{T42A} in 50 mM Bis-Tris, pH 6.5, 50 mM NaCl, 1 mM TCEP using 2D ^{1}H-^{15}N HSQC, 3D HNCACB, 3D CBCA(CO)NH. These assignments have been deposited in the Biological Magnetic Resonance Data Bank under the accession codes: 52757 (N-SH2^{WT} Apo), 52758 (NSH2^{T42A} Apo), 52759 (N-SH2^{WT} 1P-GAB1$_{N\text{-}term}$), 52760 (NSH2^{T42A} 1P-GAB1$_{N\text{-}term}$).

Backbone dynamics. ^{15}N relaxation experiments were recorded on 1.2 mM ^{15}N-labeled apo and 1P-GAB1$_{N\text{-}term}$-bound forms (1.8 mM of peptide) of N-SH2^{WT} and N-SH2^{T42A} at 600 MHz.

Transverse relaxation (R_2), longitudinal relaxation (R_1), and heteronuclear NOE measurements were recorded in an interleaved manner with the experiments from the Bruker pulse program library. For ^{15}N R_2 and R_1 rates, experiments were performed as a pseudo 3D with a relaxation delay (D1) of 2 s, SW (^{1}H) = 16 ppm with 2048 complex points, SW (^{15}N) = 35 ppm with 256 complex points in the ^{15}N dimension, and 16 transient scans (NS). The relaxation delays sampled for R_1 are *0.08, 0.16, 0.24, 0.32, 0.40, 0.56, 0.72, 0.96, 1.28, 1.68* s, with duplicate measurements at 0.16 and 0.56 s delays. T_2 delays were measured *at 0.01, 0.02, 0.03, 0.04, 0.05, 0.06, 0.07, 0.08, 0.09, 0.10, 0.11, 0.12, 0.15 s,* with duplicate measurements at 0.01 and 0.07 s delays. Experimental errors were determined to be less than 1% from duplicate measurements.

Heteronuclear ^{1}H-^{15}N NOE values were obtained from the ratio of saturated and unsaturated peak heights. Pseudo 3D experiments were carried out with relaxation delay (D1) = 5 s, SW (^{1}H) = 21 ppm with 3072 complex points in the ^{1}H dimension, and SW (^{15}N) = 28 ppm with 256 complex points, NS=32. Triplicate measurements were obtained for error analysis.

^{15}N- Carr-Purcell-Meiboom-Gill (CPMG) relaxation dispersion(6) was collected as pseudo 3Ds with a total constant CPMG relaxation period (T_{CPMG}) of 40 ms, and 14 n_{cyc} values ranging from 1 to 60. A reference experiment was acquired without a CPMG relaxation delay (n_{cyc} =0) for the calculation of $R_{2,\,eff}$ rates as a function of CPMG field strength, ν_{cpmg}. n_{cyc} of 2 and 18 are repeated measurements for error estimation. Uncertainties in $R_{2,\,eff}$ is determined based on the larger error between duplicate-based or noise-based error calculation. Each spectrum was collected with 32 scans per FID for the bound samples or 16 scans per FID for the apo samples, and a repetitive delay of 2 s.

^{1}H-^{15}N CEST(7) data was recorded at 10 Hz (for 1P-GAB1$_{N\text{-}term}$-bound N-SH2^{WT} and N-SH2^{T42A}) or 6 Hz (for apo N-SH2^{T42A}) ^{15}N B1 field strengths. B_1 field calibration was performed using the nutation method as described by ref. (8). Data was collected as a pseudo-3D with ^{15}N offsets ranging from 100 to 133 ppm

in increments of 10 Hz for both apo and 1P-GAB1$_{N\text{-term}}$-bound samples, a relaxation delay T_{relax} of 500 ms, and a reference experiment. Each spectrum was collected with 8 scans for the 1P-GAB1$_{N\text{-term}}$-bound sample and 4 scans for the apo sample, and a repetitive delay of 1.5 sec.

NMR data analysis and fitting. Peak heights for each residue were extracted from each 2D ^{15}N-HSQCs in the pseudo 3D T_1, T_2, and heteronuclear NOE experiments using POKY(9) software. R_1 and R_2 values were obtained by fitting the peak heights to a two-parameter exponential function using the RELAX software 5.0(10):

$$I(t) = I_0\, e^{-R_{1,2} * t}$$

Where I(t) is the intensity after a delay time of t and I_0 is the intensity at time t =0. Residues R4, H85, and G86 are excluded from the analysis due to overlap and signal broadening. Uncertainties in R_1 and R_2 were calculated using Monte Carlo method with 500 fit iterations.

The average heteronuclear NOE values were determined from the ratios of the peak intensities with and without proton saturation for each replicate measurement. The standard deviation of the NOE values was calculated based on the following, where NOE_i is the individual NOE value and $\overline{NOE}$ is the average NOE value:

$$s = \sqrt{\frac{1}{n-1}\sum_{i=1}^{n}(NOE_i - \overline{NOE})^2}$$

^{1}H-^{15}N CEST analysis and fitting. Peak intensities were extracted from the pseudo-3D datasets using PINT.(11) ChemEx software (https://github.com/gbouvignies/chemex)(7) was used to fit kinetic models to the data and extract the best-fit exchange parameters. Only data from 1P-GAB1$_{N\text{-term}}$-bound N-SH2^{WT} was fitted because CEST profiles from apo N-SH2^{T42A} and 1P-GAB1$_{N\text{-term}}$-bound N-SH2^{T42A} predominantly lack distinct second dips. Initially, five residues: Phe41, Thr42, Ser44, Arg46, and Thr52, which are in structured regions and have a shoulder with $|\Delta\omega_{AB}| > 0.5$ ppm, were fitted to a two-state exchange model. The exchange rate ($k_{ex,AB}$) and population (pB) obtained from this fit were fixed in the subsequent fitting of other residues to obtain the best estimates of $\Delta\omega_{AB}$. Residues that fit well with these exchange parameters are assigned to Group 1 (CEST). During the fitting process, initial estimates of $\Delta\omega_{AB}$ were provided based on the positions of the minor state dips. Due to their slower exchange rate, residues Leu43 and Ile54 were fitted into a separate group.

^{15}N CPMG analysis and fitting. Peak volumes were extracted from the pseudo-3D dataset by lineshape fitting across all 2D planes using PINT(11) software. Effective transverse relaxation rate ($R_{2,\,eff}$) was calculated at each CPMG filed strength (v_{cpmg}) using:

$$R_{2,eff} = \frac{1}{T_{CPMG}} \ln \frac{V(0)}{V(v_{cpmg})}$$

T_{CPMG} is the constant CPMG relaxation period, V(0) is the peak volume at $v_{cpmg} = 0$, $V(v_{cpmg})$ is the peak volume at each CPMG field strength (v_{cpmg}). The larger of the noise-based and duplicate-based uncertainties was used. Noise-based uncertainties were estimated from the noise level in the spectrum using PINT.(11) Duplicate-based uncertainties were calculated from two duplicate points. Relaxation

dispersion curves, $R_{2,eff}$ as a function of CPMG field strength were globally fitted using ChemEx software (https://github.com/gbouvignies/chemex).(7) ChemEx numerically integrates the Bloch-McConnell equations to simulate the evolution of the magnetization during the CPMG period and performs numerical fitting by minimizing the χ^2 value. For each data set, R_{ex} was estimated as the difference between $R_{2,eff}$ at the lowest n_{cyc} value and the average of the $R_{2,eff}$ at the last two highest n_{cyc} data points. Only residues with $R_{ex} > 1$ s^{-1} were used for global fitting. R_2 values of major and minor states were constrained to be equal. Initially, data were fitted to a 2-state exchange model and grouped based on their exchange rate. However, some probes in the 1P-GAB1$_{N\text{-}term}$ N-SH2^{WT} were not well described by a 2-state model indicating a more complex exchange process. These probes were better fitted to a bifurcated 3-state model (B↔A↔C) where A is the major, observed state and B and C are the minor states. During the 3-state fitting, the $k_{ex,AB}$, and $\Delta\omega_{AB}$ derived from ^{15}N-CEST data fitting were fixed, while *pB, pC,* $\Delta\omega_{AC}$, and $k_{ex,AC}$ were allowed to vary. The global starting value for $k_{ex,AC}$ was estimated from the 2-state fit of the ^{15}N-CPMG data for the same probes. Similarly, the pB value extracted from ^{15}N-CEST was used as the initial starting value for the 3-state global fit. We also considered the triangle model and the linear (A↔B↔C) three-state models, where A is the observed major state and B and C are minor states. Both the bifurcated model and the triangle model gave comparable fit quality (based on the χ^2_{red}, AIC, and BIC scores). However, our data do not support an exchange between the two minor states (B and C). If such exchange existed, it should have also been detected in our CPMG data for the 1P-GAB1$_{N\text{-}term}$ N-SH2^{T42A}, where the zipped B state is the major state, but we see no evidence of state B involved in fast exchange with another state C. We note that all populations estimated from CPMG fitting are underdetermined.

X-ray Crystallography

The crystallization conditions were obtained from the following commercially available screens: Crystal Screen, Index Screen, PEGIon, PegRx (Hampton Research), and JBSKinase (Jena Bioscience). Purified N-SH2 at 10 mg/mL (1:1.05 molar ratio for N-SH2:1P-GAB1$_{N\text{-}term}$ peptide) were used to set up sitting drop vapor diffusion 96 well INTELLI plates (102-0001-20, Art Robbins) using 0.5 µL of protein 0.5 µL of crystallization solution and 50 µL of the solution in the reservoir. The trays were set by a Gryphon liquid handling robot (Art Robbins), sealed using ClearVue Sheets (Molecular Dimensions), stored at 291 K, and periodically checked for crystals. Crystals grew overnight and reached final size after three days. Unbound N-SH2^{WT} was crystallized in 1.0 M sodium malonate pH 5.0, 0.1 M sodium acetate trihydrate pH 4.5, and 2% w/v polyethylene glycol 20,000; unbound N-SH2^{T42A} was crystallized in 0.1 M TRIS hydrochloride pH 8.5, 2.0 M ammonium phosphate monobasic; 1P-GAB1$_{N\text{-}term}$-bound N-SH2^{WT} : 6% v/v Tacsimate pH 6.0, 0.1 M MES monohydrate pH 6.0, and 25% w/v polyethylene glycol 4,000; 1P-GAB1$_{N\text{-}term}$-bound N-SH2^{T42A}: 0.2 M potassium sodium tartrate tetrahydrate, 0.1 M sodium citrate tribasic dihydrate pH 5.6, and 2.0 M ammonium sulfate. 10 mg/mL N-SH2^{WT} with 20 mM O-phospho-L-tyrosine (Sigma) in 0.5 M Tris pH 9.0 was used to crystallize N-SH2:phosphotyrosine complex against 100 mM sodium acetate pH 4.6, and 2 M ammonium sulfate crystallization solution.

The construct N-SH2$^{WT\text{-}short}$ and crystallization condition described in PDB entry 1AYD were reproduced. N-SH2$^{WT\text{-}short}$ was dialyzed in 10 mM MES, 50 mM KCl pH 5.5 and concentrated to 20 mg/mL. 0.5 µL protein was mixed with 0.5 µL of crystallization solution (2.05 M ammonium sulfate and 100 mM MES pH 6.0). The drop was equilibrated against 40 µL of the crystallization solution in a In Situ-1 Crystallization Plate (Mitegen) and incubated at room temperature. N-SH2$^{WT\text{-}short}$ crystals were obtained in the absence of sulfate by mixing 0.5 µL 20mg/mL protein with 0.5 µL of 1.6 M Sodium citrate tribasic dihydrate pH 6.5 using the sitting drop INTELLI plate at 293 K.

Crystals were cryoprotected either by replacing the mother liquor for Paratone® N (Hampton Research) or by bathing into a cryoprotectant solution made by mixing 3 μL of reservoir solution with 1 μL of 100% glycerol (Hampton Research). The cryoprotected crystals were harvested in 18 mm Mounted CryoLoops™ (Hampton Research), flash-cooled in liquid nitrogen, and shipped to the synchrotron X-ray facility.

Data collections were carried out cryogenically at the Advanced Light Source (beamlines 8.2.1 and 5.0.2), Stanford Synchrotron Radiation Lightsource (beamlines 12-1 and 14-1), and National Synchrotron Light Source II (beamline 17-ID-1).(12) Diffraction images were processed in XDS(13) or iMosflm(14), scaled, and merged in Aimless with the resolution cutoff set to $CC_{0.5}$ higher than 0.3.(15) The structures were solved by molecular replacement using the PDB entry 4qsy as the search model in Phaser.(16) The model refinement was conducted in *phenix.refine(17, 18)* with manual building in Coot(19) and model quality assessed using Molprobity.(20) The refined model was used as input in *phenix.ensemble_refinement* with a grid scan for the parameters wxray, pTLS, and tx.(21) The ensemble refinement combination of parameters that resulted in the lowest R-free was chosen for the final ensemble model. PyMOL was used for model visualization, interpretation, and preparation of figures.

Structure prediction

Predictions of full-length WT and E139D SHP2 structures bound to BTLA were obtained using Alphafold multimer v3 colab notebook using dropout, 12 recycles, and 8 seeds.(22) The resulting structures in the open state were aligned and visualized in PyMOL.

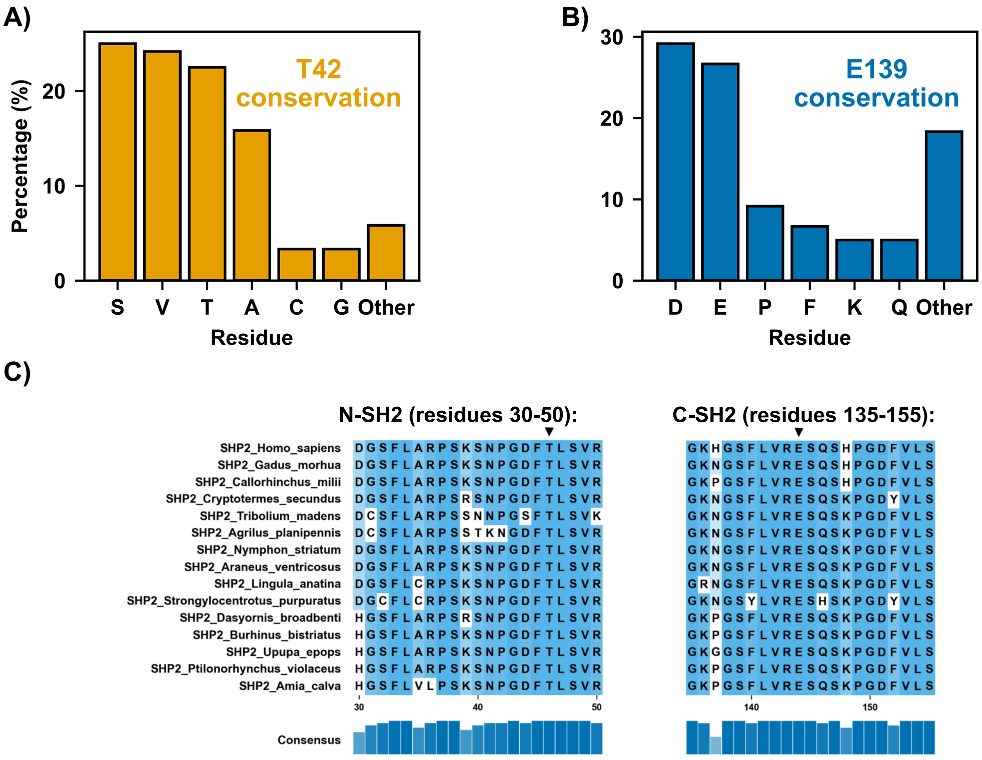

Fig. S1. Thr42 and Glu139 are poorly conserved among human SH2 sequences, but highly conserved among SHP2 and its orthologs. (A, B) Conservation analysis of residues in human SH2 domains corresponding to Thr42 and Glu139, respectively, based on a multiple sequence alignment from the SH2 database (SH2db).(23) The six most common residues at each position are shown as percentages, revealing poor conservation at both sites. **(C)** Sequence alignment of N-SH2 and C-SH2 domains of SHP2 across various species. Relevant N-SH2 and C-SH2 sites are shown. The mutation sites Thr42 and Glu139 are marked with black arrows, illustrating their full conservation across orthologs.

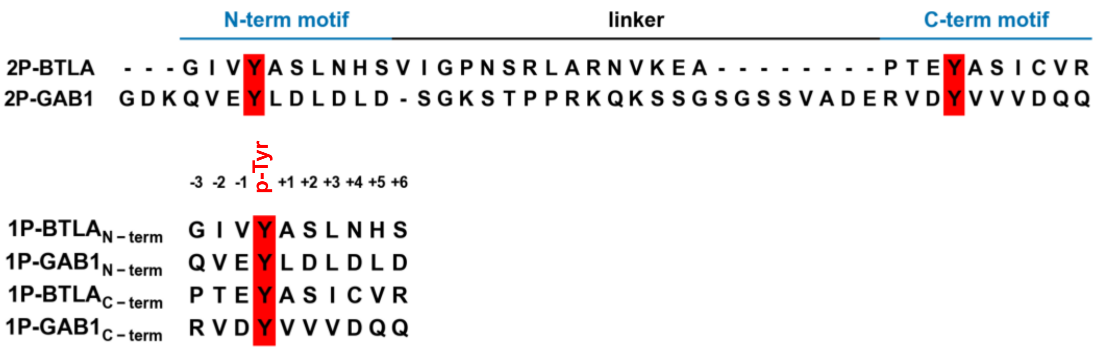

Fig. S2. Sequences of the activating phosphopeptides BTLA and GAB1 used in this study. On top, the full, bis-phosphorylated (2P) peptides are shown, used in activity assays. The singly phosphorylated (1P) N-terminal (N-term) and C-terminal (C-term) phosphotyrosine (p-Tyr) peptides used in this study are shown below. p-Tyr residues are highlighted in red.

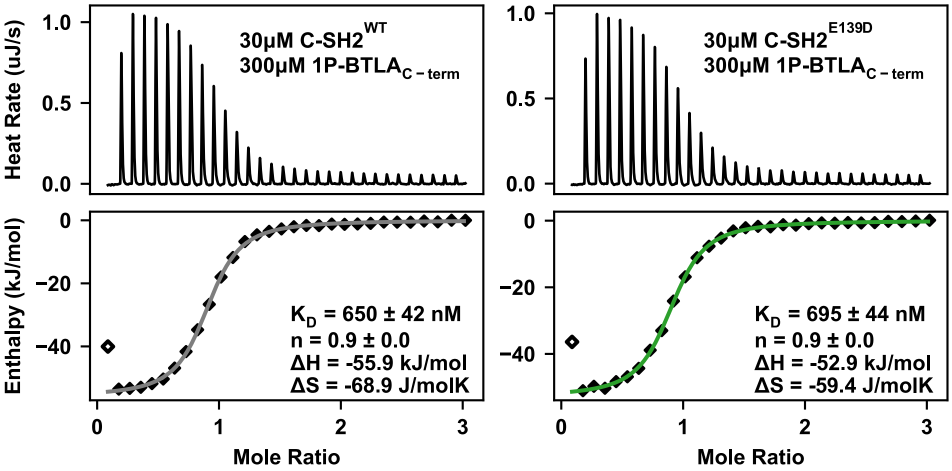

Fig. S3. The E139D mutation does not increase C-SH2 phosphopeptide binding affinity Isothermal titration calorimetry (ITC) data comparing the binding of 1P-BTLA$_{\text{C-term}}$ to C-SH2^{WT} (left) and C-SH2$^{\text{E139D}}$ (right). The ITC curves reveal no significant difference in binding affinity between WT and E139D variants, indicating that the E139D mutation does not alter the intrinsic phosphopeptide binding properties of the C-SH2 domain. Concentrations of protein and peptide are displayed in the plots.

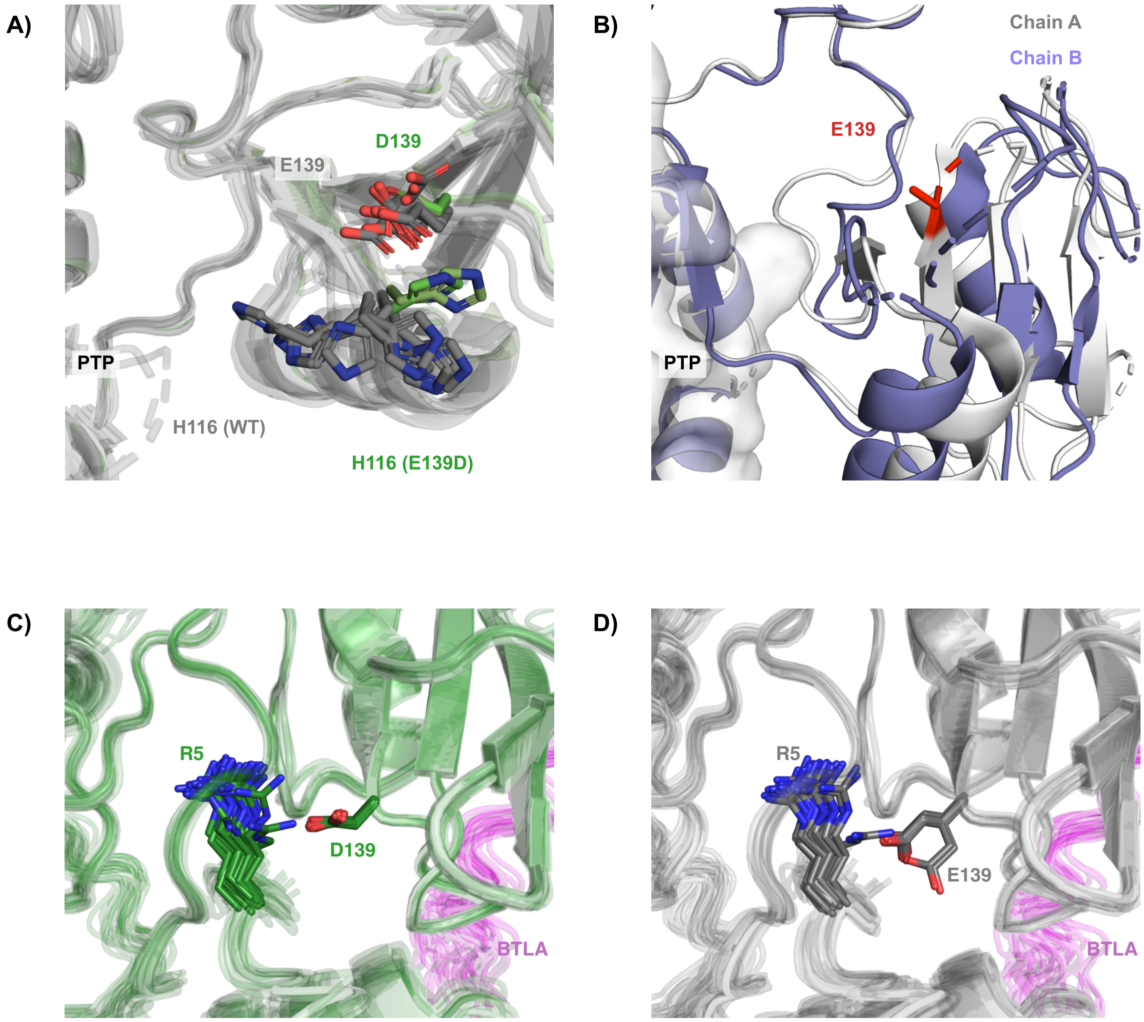

Fig. S4. **Hypotheses of how E139D mutation could shift the equilibrium to the open conformation**. **(A)** His116 occupies a wide range of conformational substates across closed SHP2 structures. Compared to WT SHP2 (grey), His116 in the E139D closed structure (green) is further shifted away from the PTP/C-SH2 interface. **(B)** X-ray structure of SHP2 (E76K variant) in the open conformation. The electron density for Glu139 (and the p-Tyr binding loop) is weak in both chains of the crystal structure, suggesting Glu139 is dynamic in the open state. (C,D) Alphafold2 predictions of the open state E139D SHP2 (green) bound to BTLA (magenta) (C) indicate that Asp139 can interact with Arg5, potentially stabilizing the open conformation, and that The Glu139 in WT SHP2 (grey) (D), possessing a longer side chain, samples more rotameric states possibly leading to a weaker interaction. These observations offer plausible hypotheses of how the E139D mutation may destabilize the closed conformation and/or stabilize the open conformation.

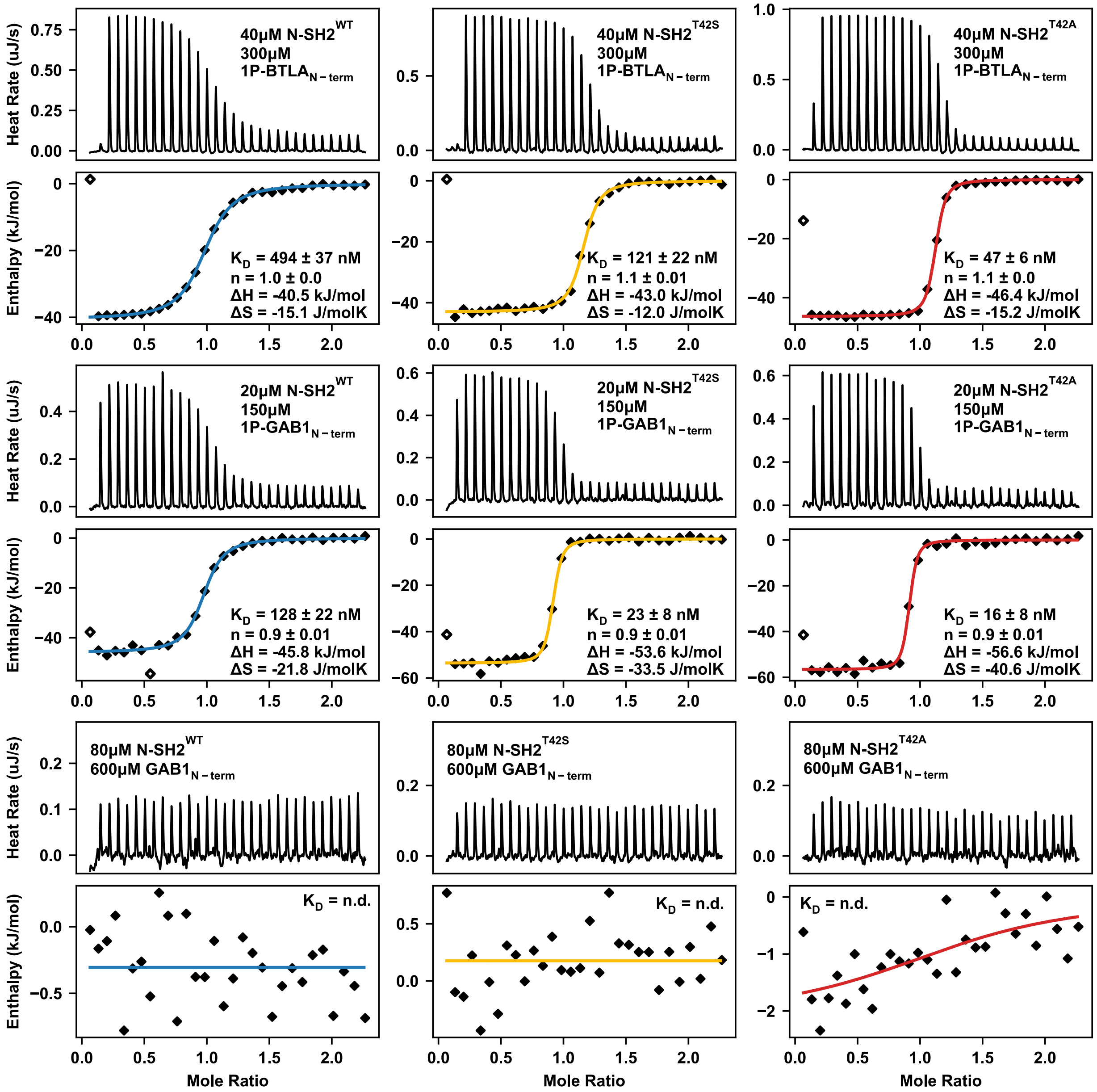

Fig. S5. The T42A mutation increases N-SH2 phosphopeptide binding affinity. ITC analysis of N-SH2 variants binding to phosphopeptide ligands. Binding affinities are compared for N-SH2^{WT}, N-SH2^{T42S}, and N-SH2^{T42A} variants for three ligands: 1P-BTLA$_{N\text{-term}}$ (top), 1P-GAB1$_{N\text{-term}}$ (middle), and the unphosphorylated GAB1$_{N\text{-term}}$ (bottom). The T42A mutation significantly increases binding affinity for phosphorylated ligands. Concentrations of protein and peptide are displayed in the plots.

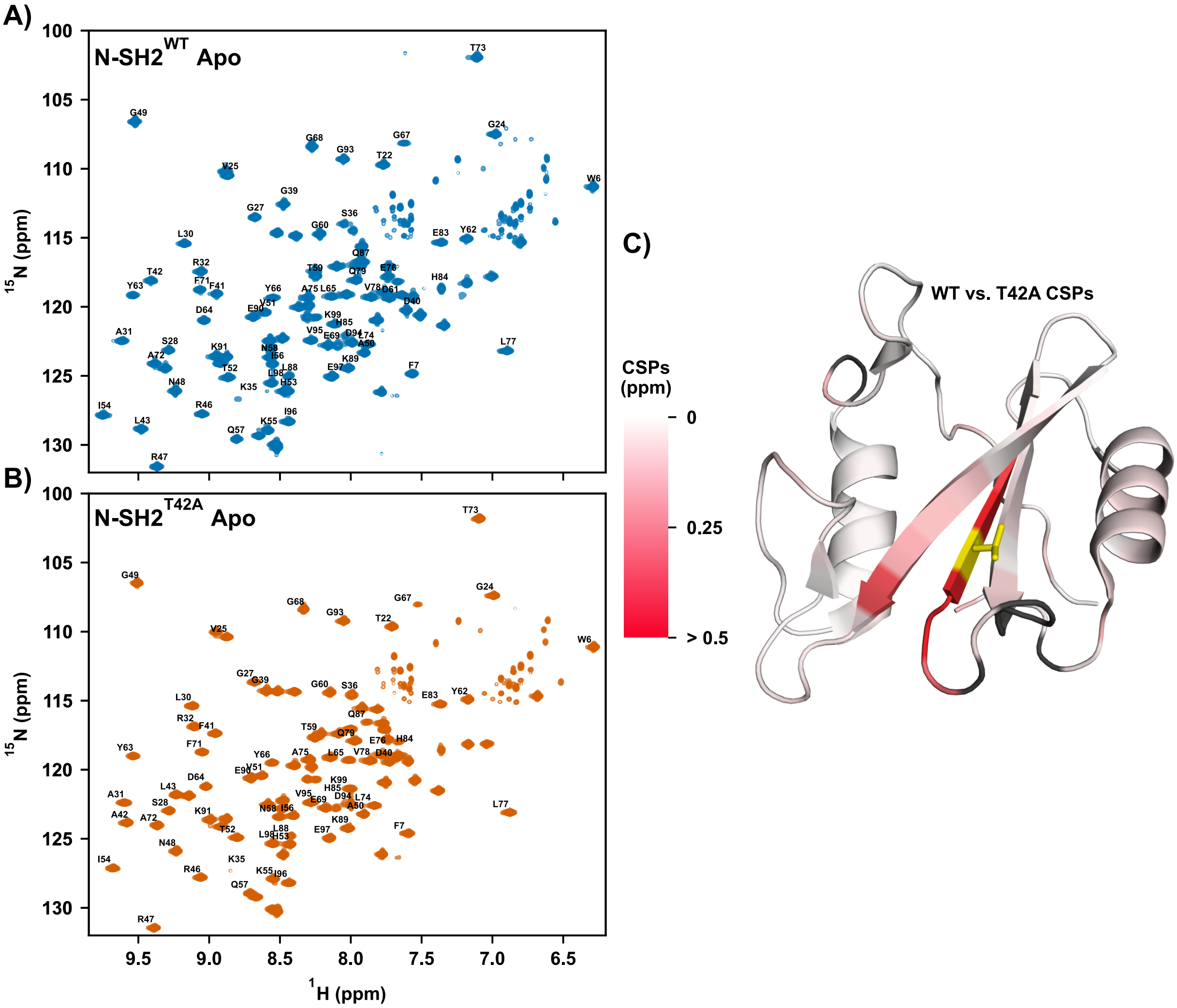

Fig. S6. The T42A mutation induces global chemical shift perturbations. (A) ^{1}H-^{15}N TROSY-HSQC spectra of SHP2 N-SH2^{WT} in the apo state. **(B)** ^{1}H-^{15}N TROSY-HSQC spectra of SHP2 N-SH2^{T42A} in the apo state. **(C)** Chemical shift perturbations comparing apo N-SH2^{WT} and N-SH2^{T42A} plotted onto the apo N-SH2^{WT} structure with the site of mutation (Thr42) shown in yellow.

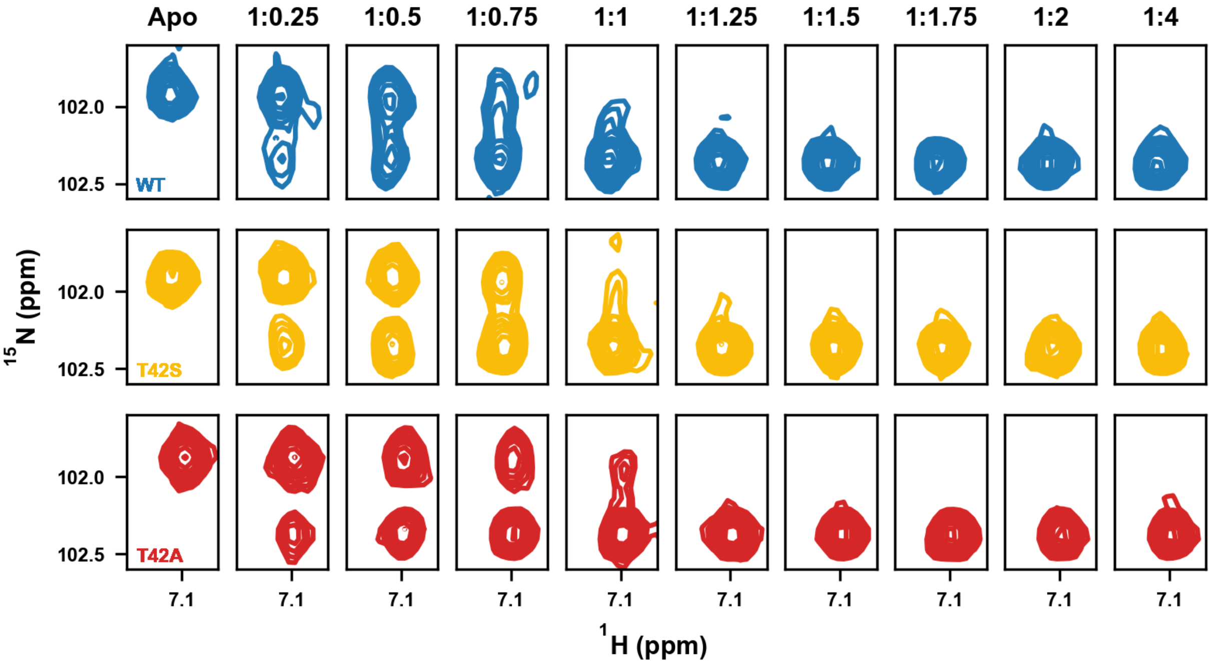

Fig. S7. Excess peptide relative to protein resulted in no additional peak shifts. Zoom in of residue Thr73 in ^{1}H-^{15}N TROSY-HSQC spectra of 100 µM ^{15}N labeled SHP2 N-SH2^{WT}, N-SH2^{T42S}, and N-SH2^{T42A} titrating in 1P-BTLA$_{N\text{-term}}$, with the molar ratios of peptide to protein displayed above the plots.

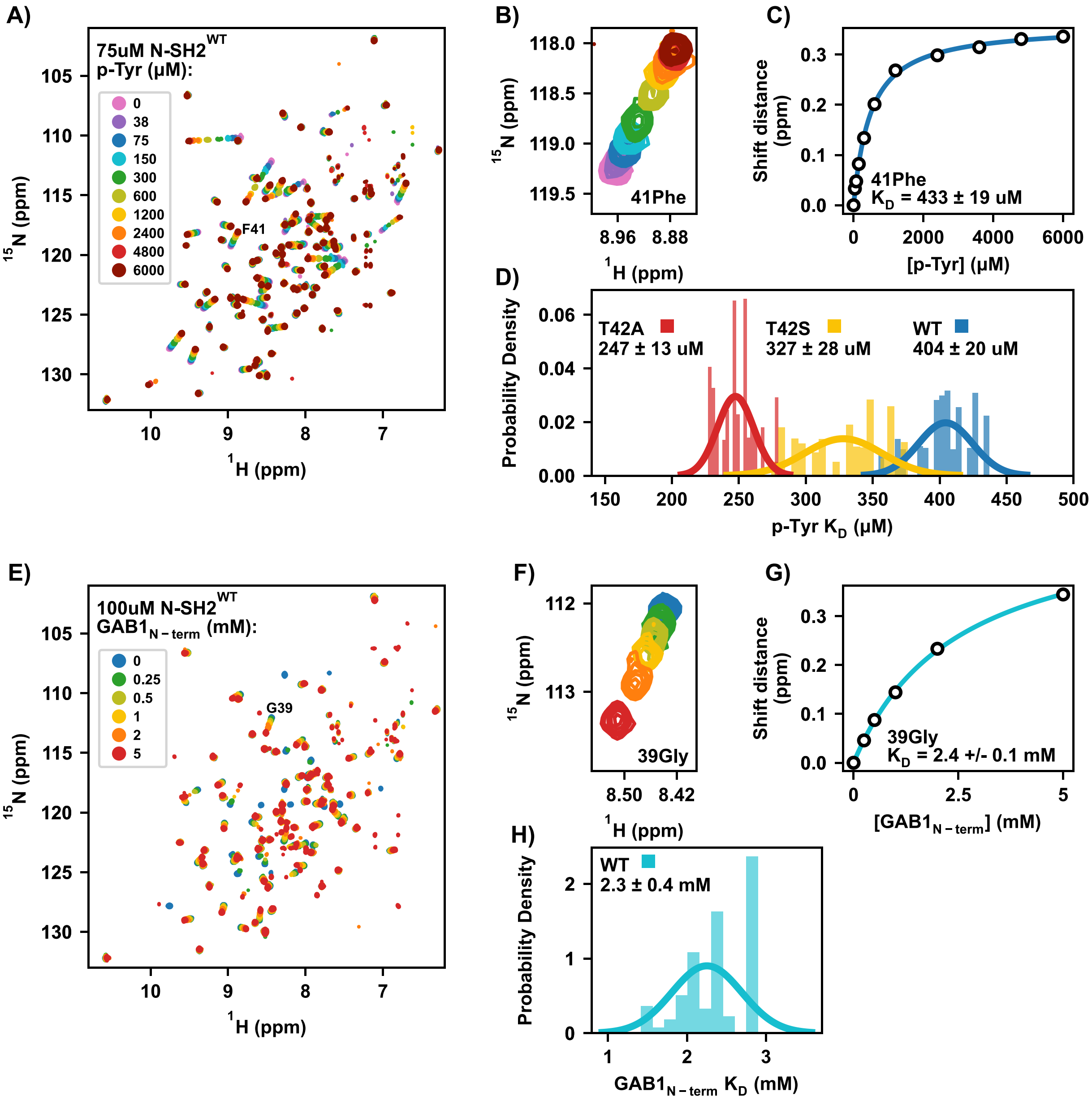

Fig. S8. Phosphotyrosine binding and unphosphorylated peptide binding to N-SH2 is weak. (A) ^{1}H-^{15}N TROSY-HSQC spectrum of N-SH2^{WT} upon binding of increasing amounts of phosphotyrosine (p-Tyr). **(B)** Zoom-in of **(A)**, showing the amide peak of Phe41. **(C)** Quantification of the chemical shift changes shown in **(B)** and corresponding fit to extract the K_D value. **(D)** Distribution of K_D values extracted from each chemical shift perturbation for p-Tyr titrations of N-SH2^{WT}, N-SH2^{T42S}, and N-SH2^{T42A} revealing a 1.6-fold tighter binding for N-SH2^{T42A} relative to N-SH2^{WT}. **(E)** ^{1}H-^{15}N TROSY-HSQC spectrum of N-SH2^{WT} upon binding of increasing amounts of unphosphorylated GAB1$_{N\text{-term}}$. **(F)** Zoom-in of **(E)**, showing the amide peak of Gly39. **(G)** Quantification of the chemical shift changes shown in **(F)** and corresponding fit to extract the K_D value. **(H)** Distribution of K_D values extracted from each chemical shift perturbation for peptide titrations of N-SH2^{WT}.

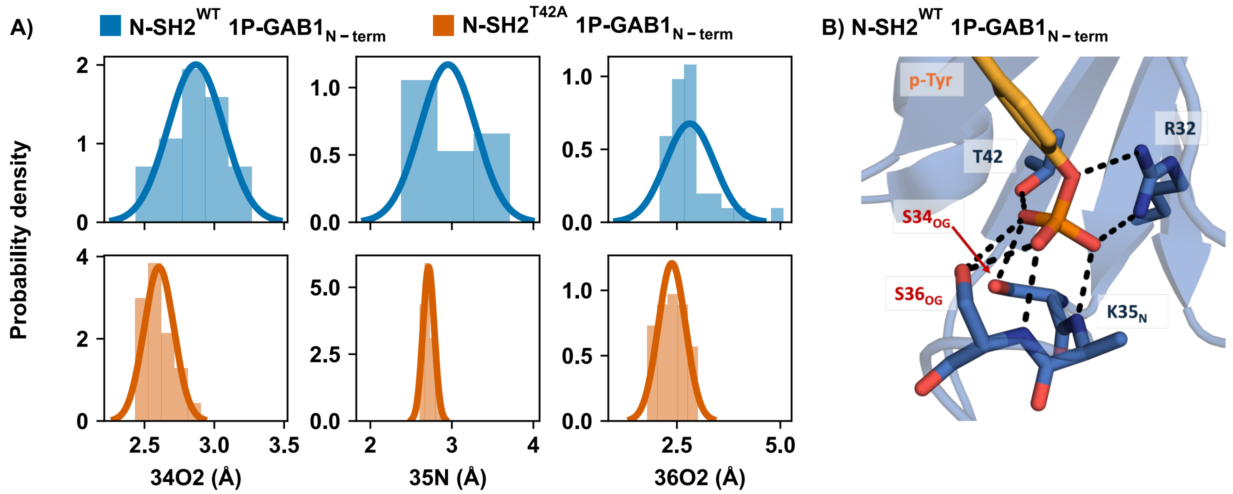

Fig. S9. **Structural hypothesis for explaining the slightly increased p-Tyr binding affinity of N-SH2^{T42A} relative to N-SH2^{WT}. (A)** Distances of key residues in the N-SH2 phosphotyrosine binding loop to the phosphate oxygens of bound 1P-GAB1$_{N\text{-term}}$ from the ensemble refined X-ray crystal structures of N-SH2^{WT} and N-SH2^{T42A}. The sidechain hydroxyls of Ser34 (O2), Ser36 (O2), and the amide backbone of Lys35 are closer to the phosphate group in N-SH2^{T42A} compared to N-SH2^{WT}, indicating enhanced stabilization of the p-Tyr interaction. These interactions, as well as those of Arg32 and Thr42 are highlighted in **(B)** our X-ray crystal structure of N-SH2 WT bound to 1P-GAB1$_{N\text{-term}}$. These shorter distances likely contribute to the increased binding affinity of T42A for p-Tyr that we measured (see **Fig. 2F**).

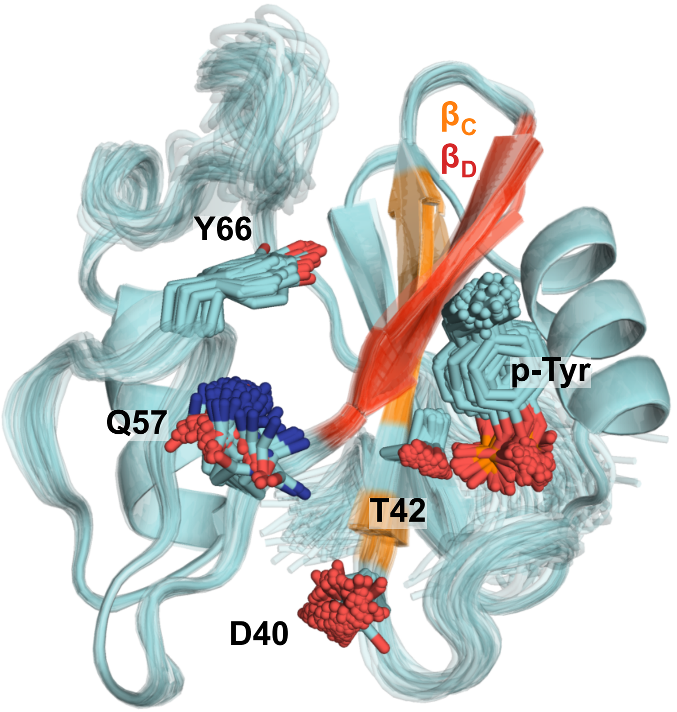

Fig. S10. **Ensemble refinement of X-ray crystal structure of N-SH2^{WT} bound to p-Tyr alone**. The beta-strands are in the unzipped conformation, with Tyr66 adopting a position that blocks the hydrophobic cleft required for full peptide binding, and Gln57 occupies a position that prevents the formation of a stabilizing Tyr66-Asp40 hydrogen bond. A shift in Gln57's position is required for this interaction to occur. This structure supports a multi-step binding mechanism, where initial p-Tyr binding occurs in an unzipped conformation, followed by conformational rearrangements to accommodate the complete phosphopeptide binding.

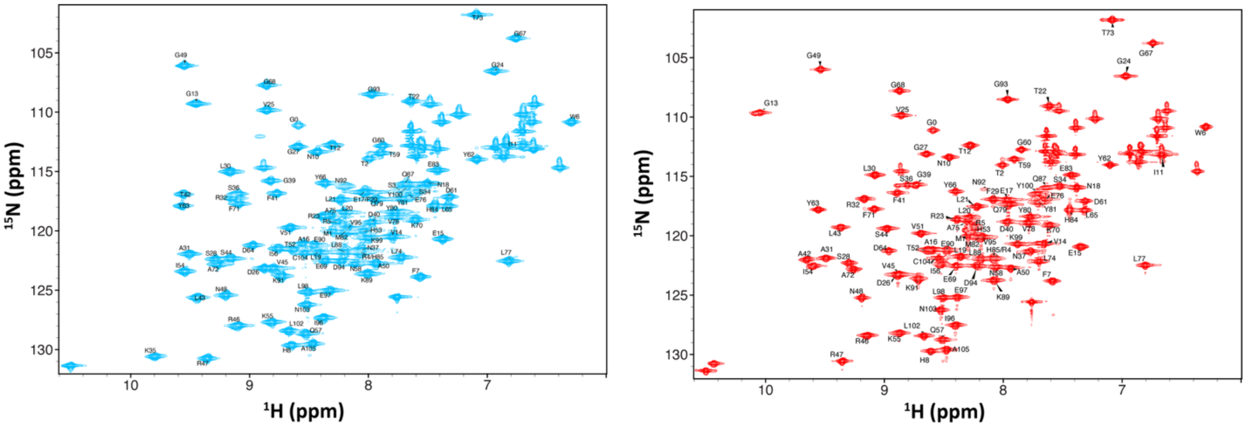

Fig. S11. ^{1}H-^{15}N TROSY-HSQC spectra of SHP2 N-SH2^{WT} (left) and N-SH2^{T42A} (right) bound to 1P-GAB1$_{N\text{-}term}$.

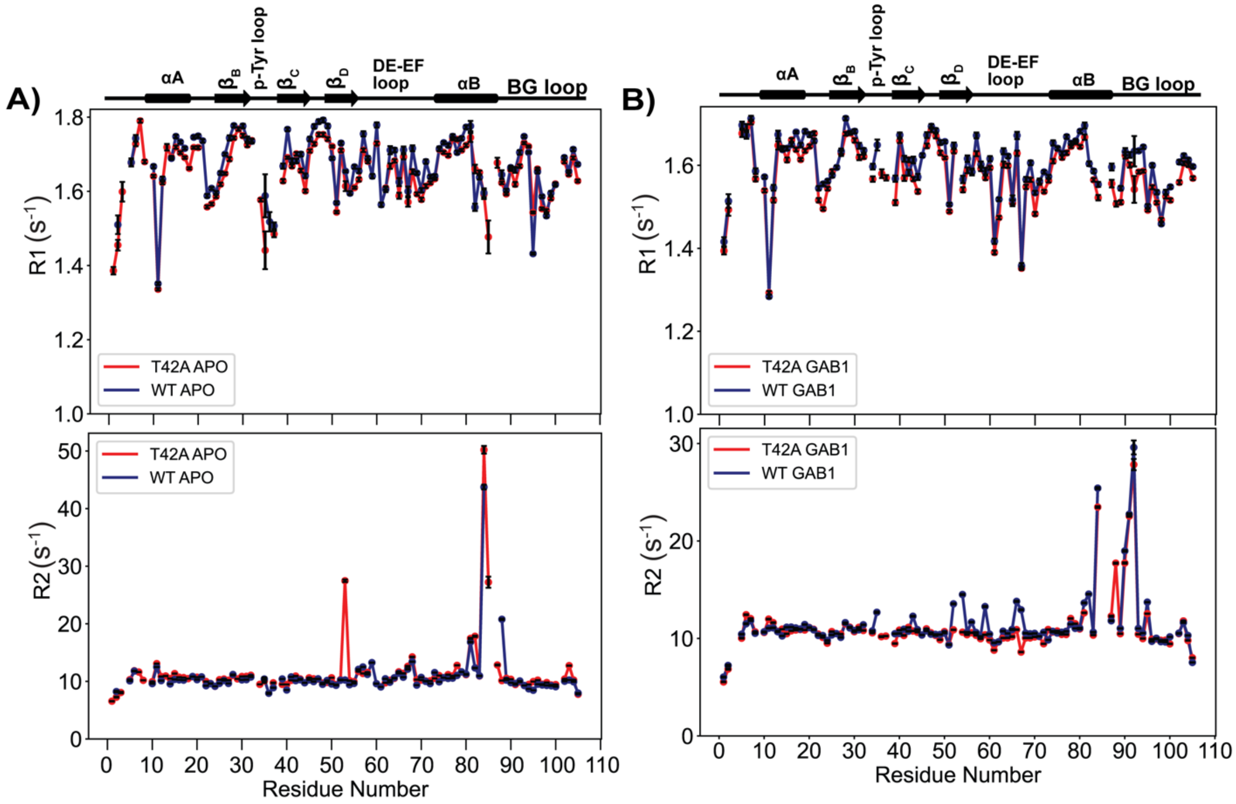

Fig. S12. ^{15}N R_1, and R_2 plots for the apo, and the 1P-GAB1$_{N\text{-}term}$ bound forms of the N-SH2 variants. **(A)** The top panel shows the overlays of R_1 relaxation values of apo N-SH2 variants, and the bottom panel shows the overlays of R_2 relaxation values of apo N-SH2 variants. **(B)** The top panel shows the overlays of R_1 relaxation values of the 1P-GAB1$_{N\text{-}term}$-bound (denoted GAB1 in the figure labels) N-SH2 variants, and the bottom panel shows the overlays of R_2 relaxation values of the 1P- GAB1$_{N\text{-}term}$-bound N-SH2 variants. Missing data points are from proline residues and overlapped residues. All datasets were collected at 25 °C.

A

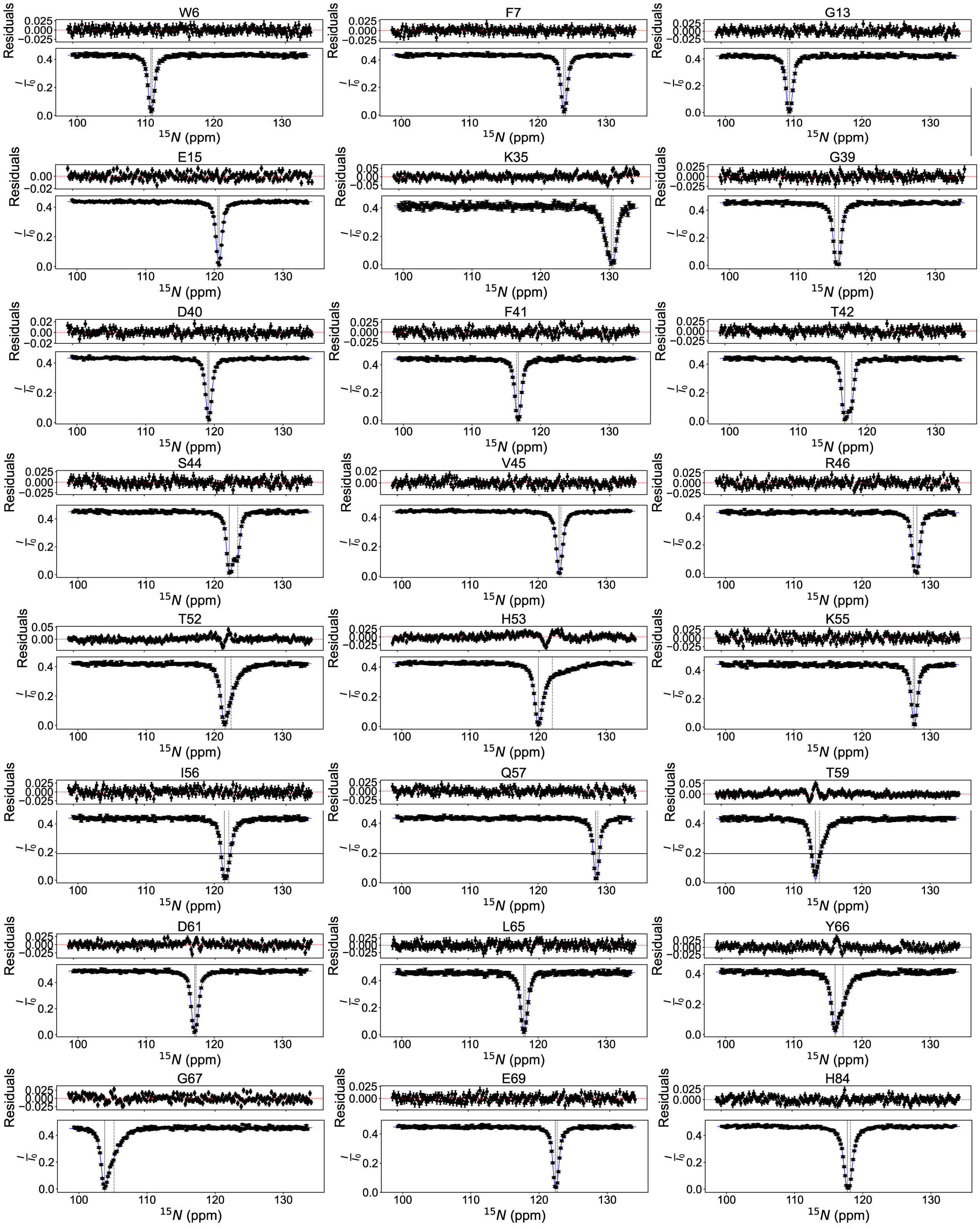
W6
F7
G13
E15
K35
G39
D40
F41
T42
S44
V45
R46
T52
H53
K55
I56
Q57
T59
D61
L65
Y66
G67
E69
H84
Residuals
$\frac{I}{I_0}$
^{15}N (ppm)

A) (CONT'D)

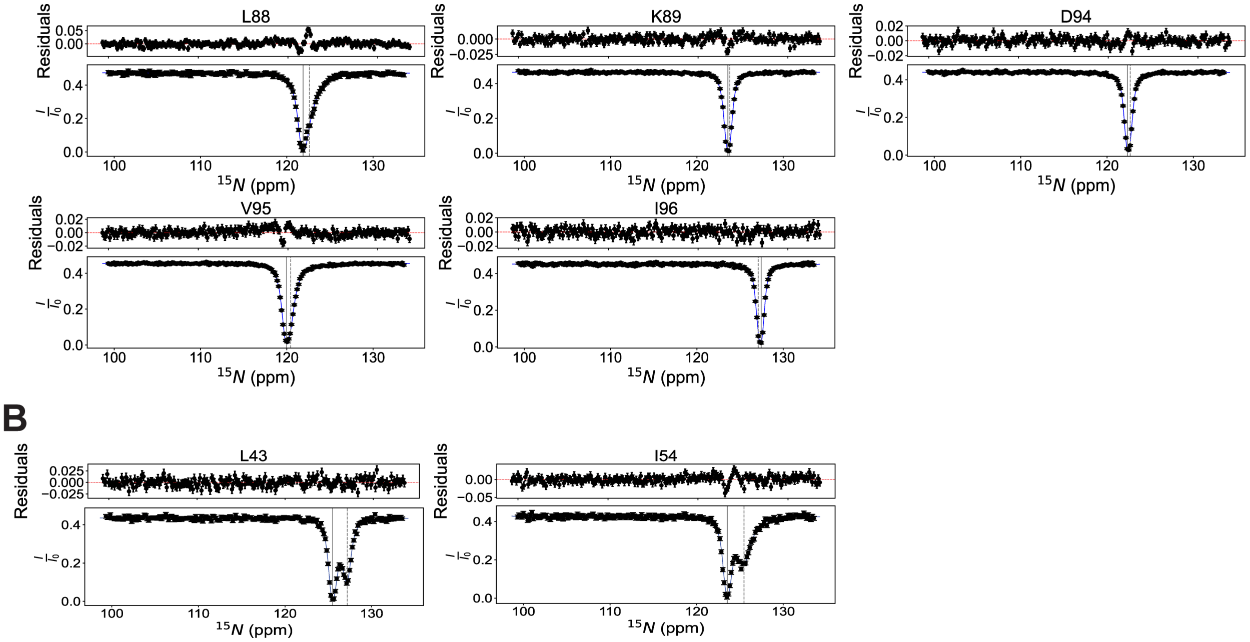

Fig. S13. ^{15}N CEST profiles of group-fitted residues in 1P-GAB1$_{\text{N-term}}$ bound N-SH2^{WT}. (A) ^{15}N CEST profiles of residues fitted to a 2-state exchange resulting in k_{ex} = 104 ± 8.5 s^{-1}, pB = 11 ± 0.3 %. **(B)** ^{15}N CEST profiles residues fitted to a 2-state exchange process resulting in k_{ex} = 45 ± 5 s-1, pB = 16 ± 0.6 %. Data were collected with 10 Hz weak B_1 field strength and 10 Hz spacing between the offsets, at 25 °C. The solid black line indicates the fit, the gray solid line indicates the major state position, and the dashed gray line indicates the minor state position.

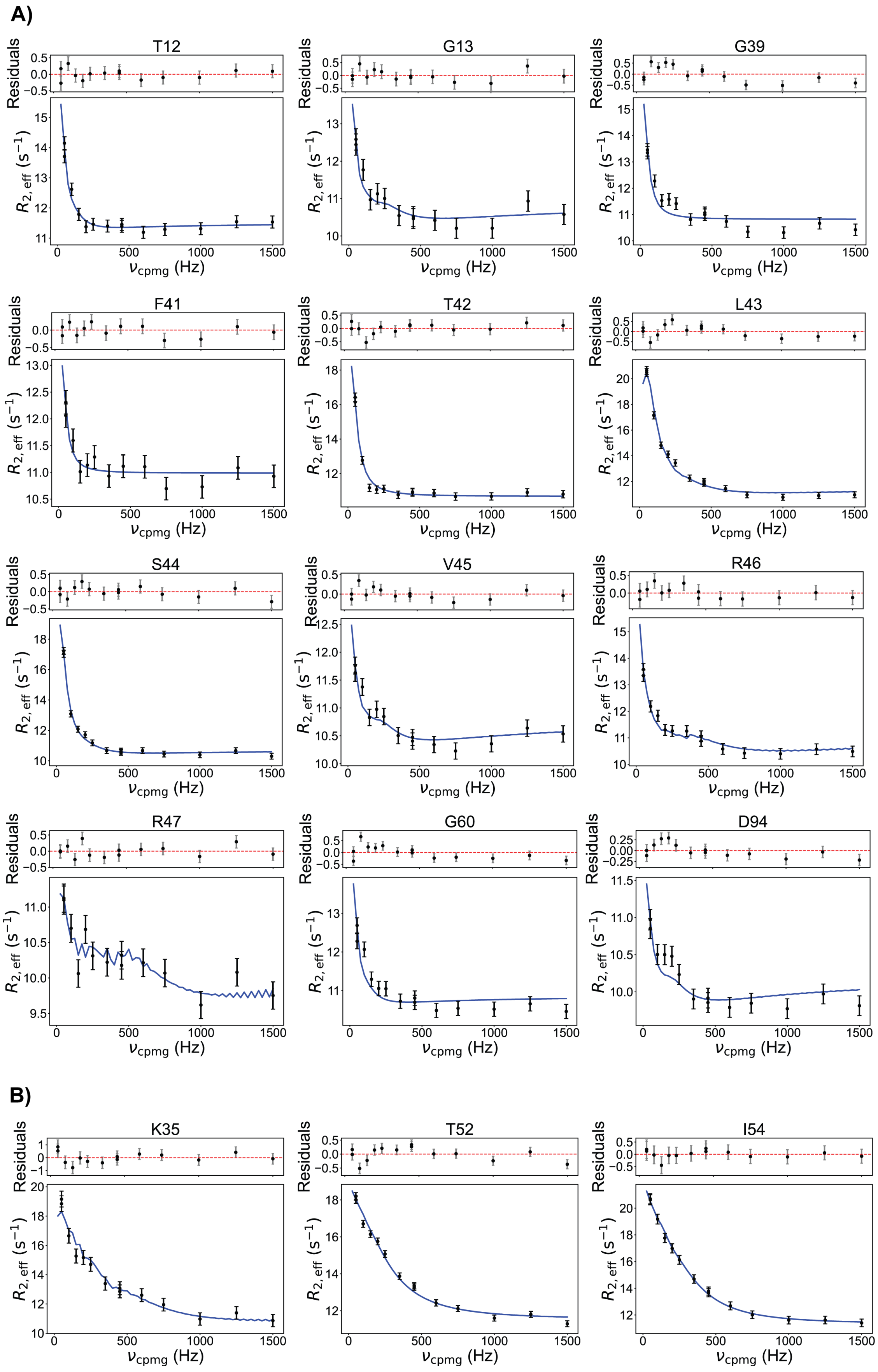
A)
T12
G13
G39
F41
T42
L43
S44
V45
R46
R47
G60
D94
B)
K35
T52
I54
Residuals
$R_{2,\mathrm{eff}}$ (s^{-1})
ν_{cpmg} (Hz)

B) (CONT'D)

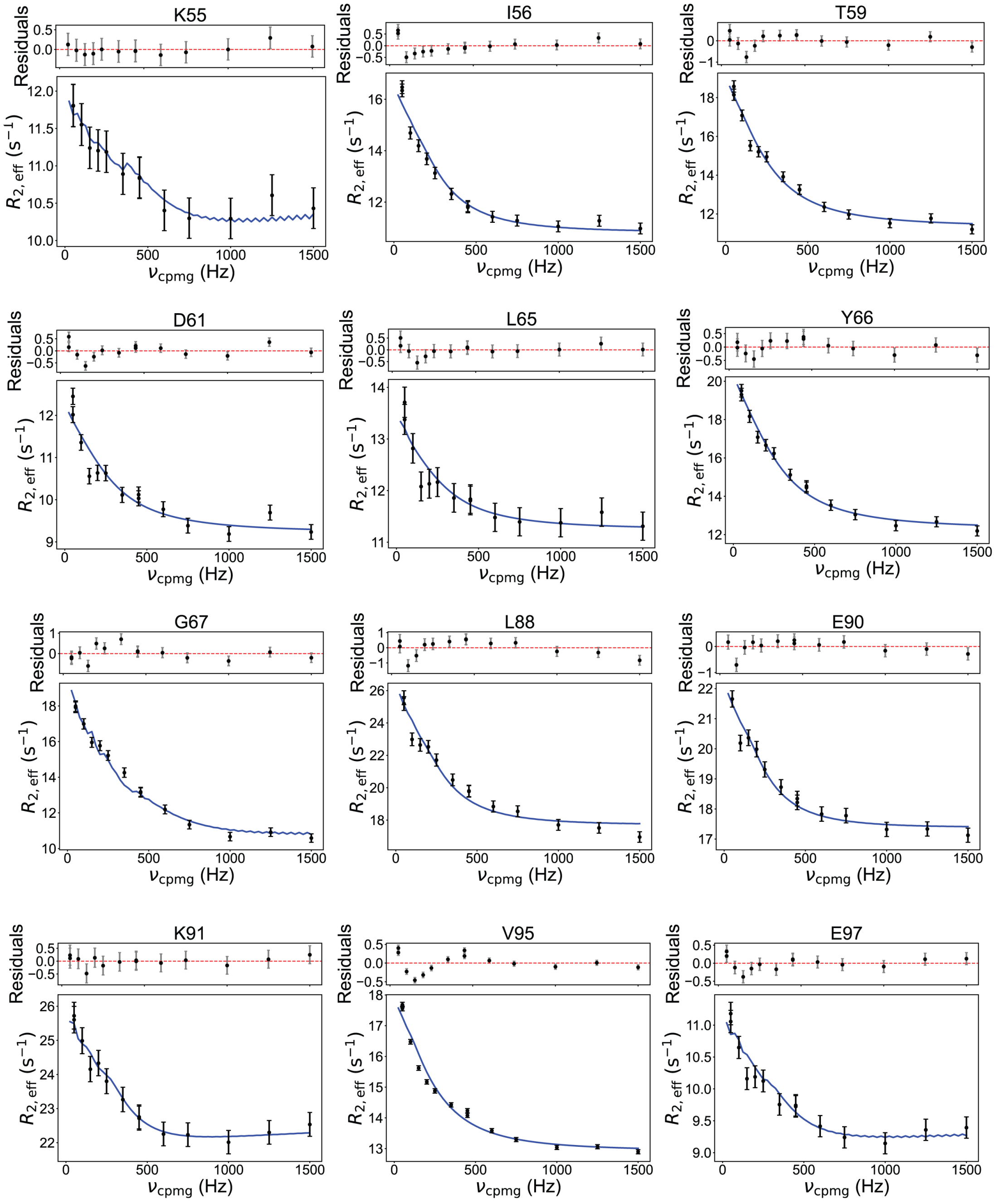

K55
I56
T59
D61
L65
Y66
G67
L88
E90
K91
V95
E97
Residuals
$R_{2,\mathrm{eff}}$ (s^{-1})
ν_{cpmg} (Hz)

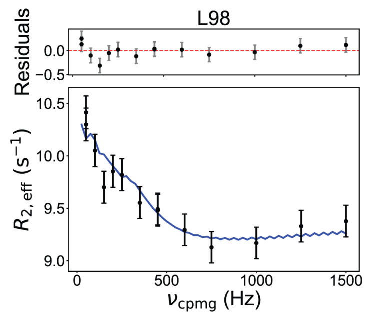

L98
Residuals
$R_{2,\mathrm{eff}}$ (s^{-1})
ν_{cpmg} (Hz)

C)

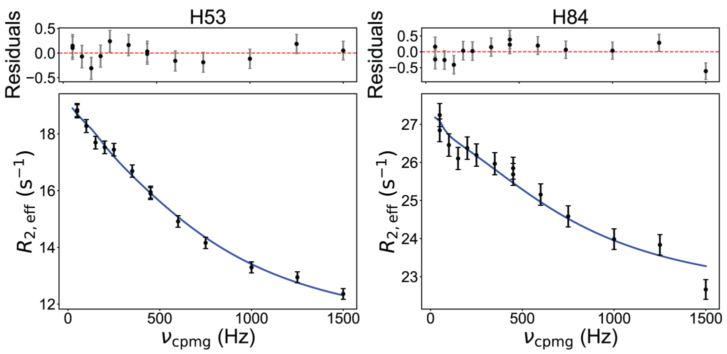

H53
H84
Residuals
$R_{2,\mathrm{eff}}$ (s^{-1})
ν_{cpmg} (Hz)

Fig. S14. ^{15}N CPMG profiles of all residues in 1P-GAB1$_{\text{N-term}}$ bound N-SH2^{WT} group fitted to 2-state exchange. (A) Group 1 residues (k_{ex} = 104 ± 8.5 s^{-1}; pB = 11 ± 0.3 %) with χ^2_{red}, AIC, BIC = 1.6, 96, 164. (**B**) Group 2 residues (k_{ex} = 1740 ± 100 s^{-1}) with χ^2_{red}, AIC, BIC = 2.4, 242, 360. **(C)** Group 3 residues (k_{ex} = 5000 ± 750 s^{-1}). All datasets were collected with a 40 ms relaxation delay and at 25 °C.

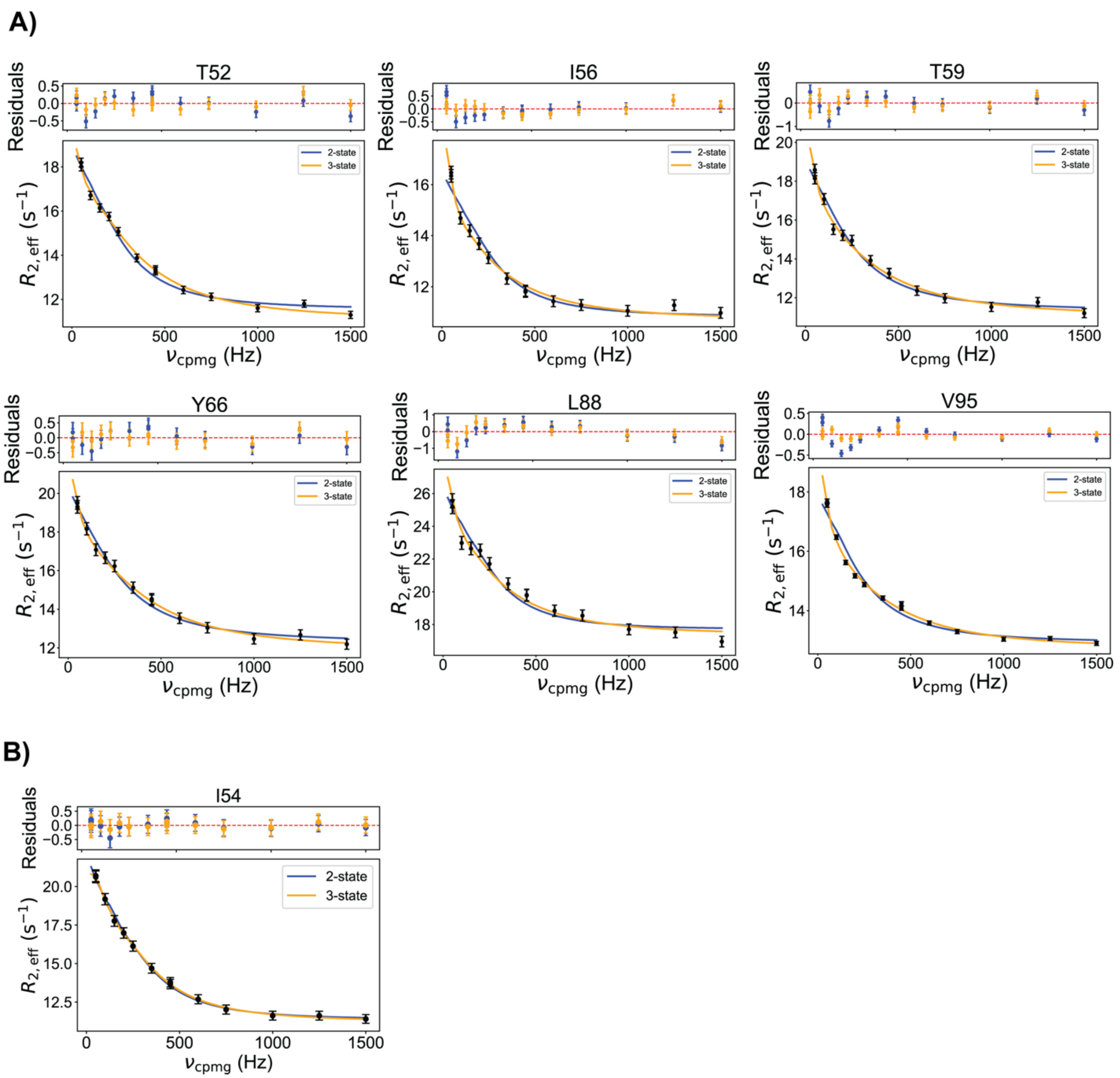

Fig. S15. Overlay of 1P-GAB1$_{\text{N-term}}$ bound N-SH2^{WT} CPMG data fits to 2-state (blue curve) versus 3-state exchange models (orange curve). (A) ^{15}N CPMG profiles of the six residues in Group 2 (**Fig. S13B**) that needed to be fit to a 3-state exchange model described in **Fig. 5F** (orange curve), resulting in $k_{ex,AC}$ = 2600 ± 100 s^{-1}, underdetermined *pB* = 3.6 ± 0.3 %, *pC* = 43 ± 6.3 % with χ^2_{red}, AIC, BIC = 1.2, 29, 67 (compared to χ^2_{red}, AIC, BIC = 2.4, 242, 360 in the 2-state fitting). The $k_{ex,AB,}$ 104 ± 8.5 s^{-1}, and $\Delta\omega_{AB}$ were fixed during the fitting process using ^{15}N CEST parameters. **(B)** 3-state fitting of the ^{15}N CPMG profile of residue Ile54 yielded a $k_{ex,AC}$ = 1830 ± 400 s^{-1} for the exchange between states A and C as described in the main text. The $k_{ex,AB}$ (45 ± 5 s^{-1}), and $\Delta\omega_{AB}$ obtained from ^{15}N CEST were fixed during the fitting process. All datasets were collected with a 40 ms relaxation delay and at 25 °C. For residues requiring three-state fitting, slow A⟷B parameters were fixed to CEST-derived values; fast-exchange populations remain underdetermined.

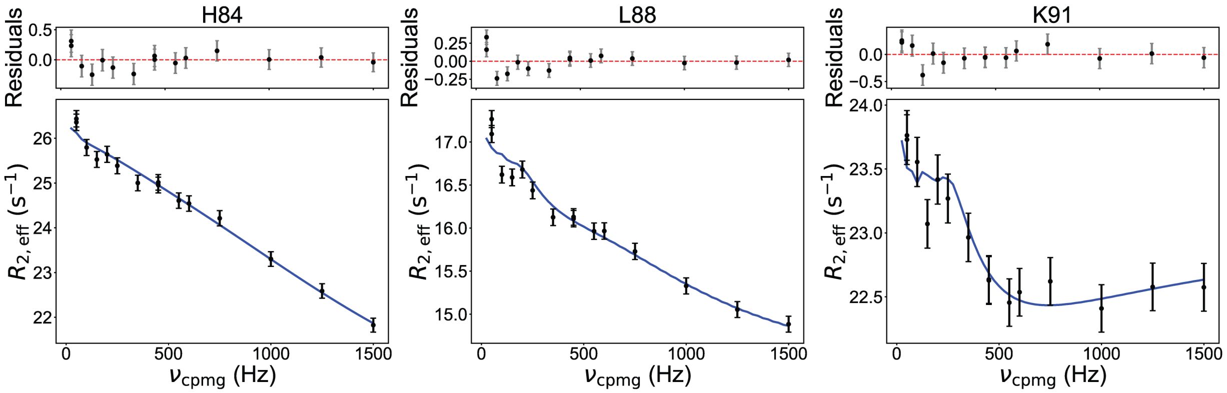

Fig. S16. 2-state exchange fits of 1P-GAB1$_{N\text{-}term}$-bound N-SH2^{T42A} ^{15}N CPMG data. ^{15}N CPMG profiles of residues in 1P-GAB1$_{N\text{-}term}$-bound N-SH2^{T42A} that show dispersion with $R_{ex} > 1$ s^{-1} but could not be fitted to a group (colored brown in **Fig. 5J**). All datasets were collected with a 40 ms relaxation delay and at 25 °C.

A)

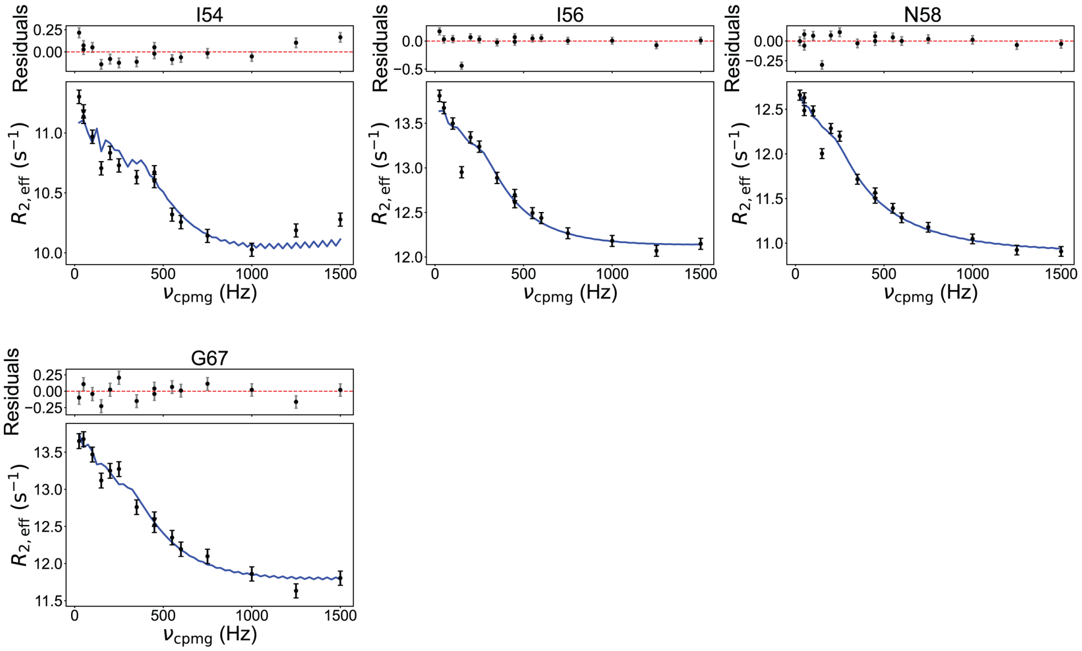
I54
I56
N58
G67
Residuals
$R_{2,\mathrm{eff}}$ (s^{-1})
ν_{cpmg} (Hz)

B)

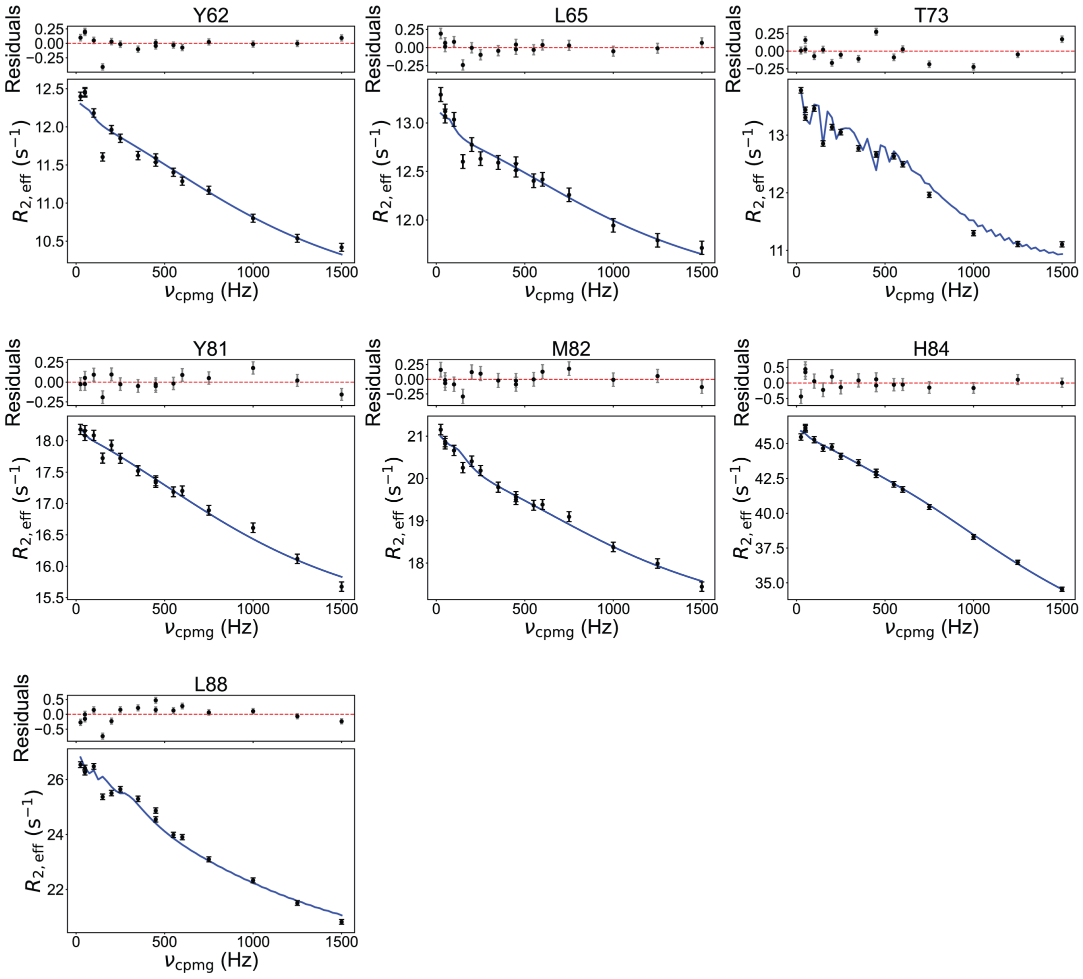
Y62
L65
T73
Y81
M82
H84
L88
Residuals
$R_{2,\mathrm{eff}}$ (s^{-1})
ν_{cpmg} (Hz)

C)

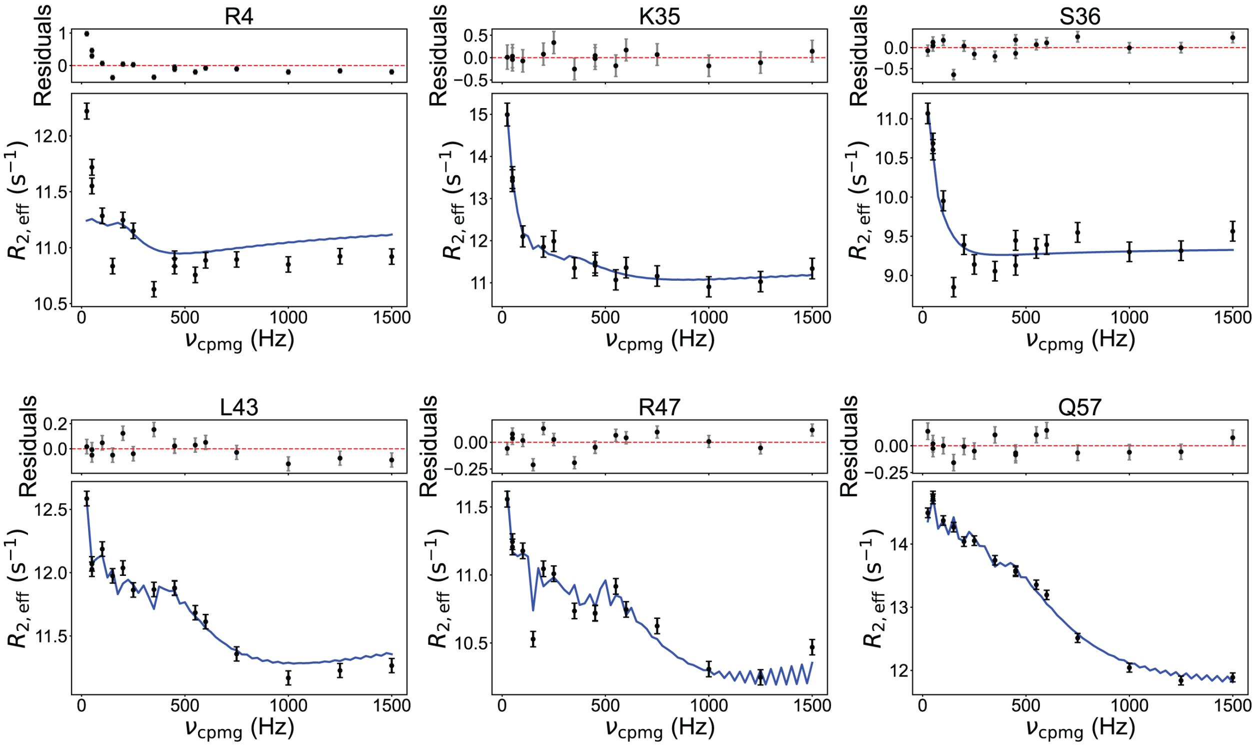

Fig. S17. **^{15}N CPMG profiles of residues in apo N-SH2^{WT} fitted to 2-state exchange. (A)** ^{15}N CPMG profiles of residues fitted to a 2-state exchange resulting in k_{ex} = 3550 ± 320 s^{-1}. **(B)** ^{15}N CPMG profiles of residues fitted to a 2-state exchange resulting in k_{ex} = 7000 ± 670 s^{-1}. **(C)** Profiles of residues in apo N-SH2^{WT} that could not be fitted to a group (colored brown in **Fig. 5K**). These are highly dynamic exchange processes between two states, A and D. All datasets were collected with a 40 ms relaxation delay and at 25 °C.

A)

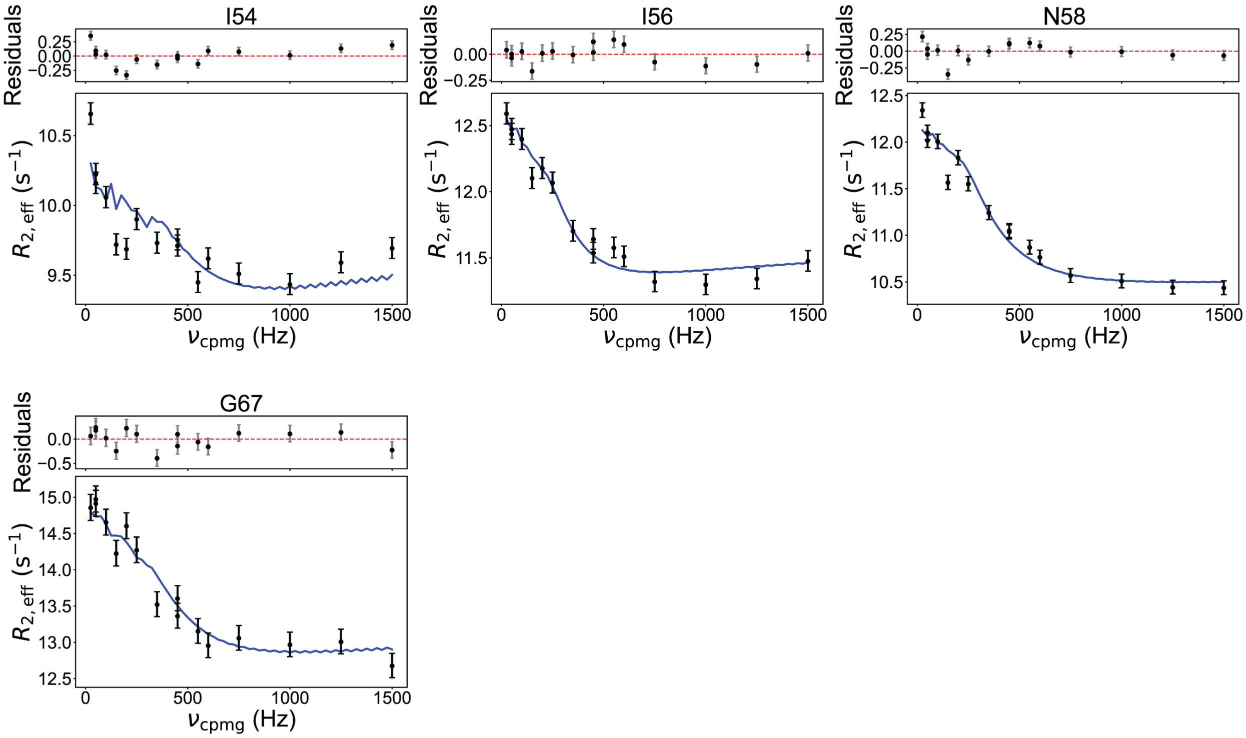
I54
I56
N58
G67
Residuals
$R_{2,\mathrm{eff}}$ (s^{-1})
ν_{cpmg} (Hz)

B)

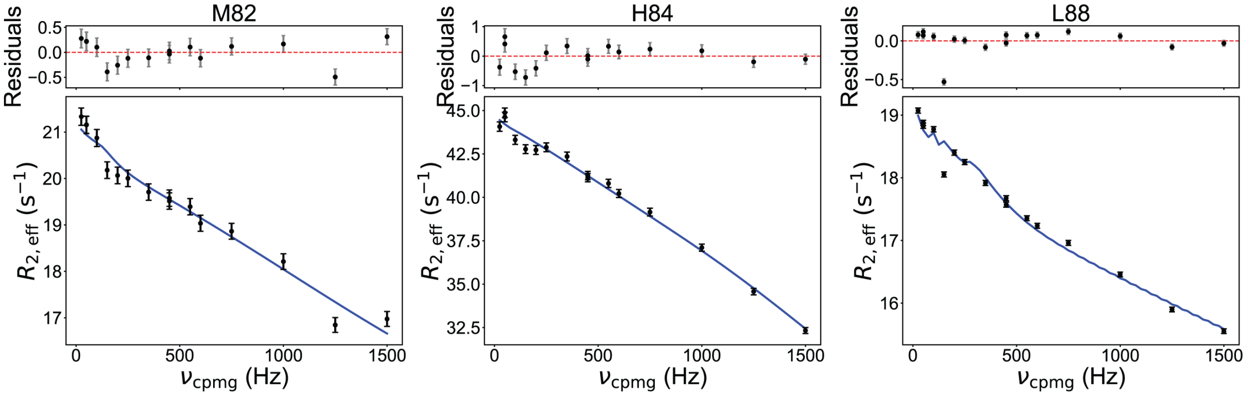
M82
H84
L88
Residuals
$R_{2,\mathrm{eff}}$ (s^{-1})
ν_{cpmg} (Hz)

C)

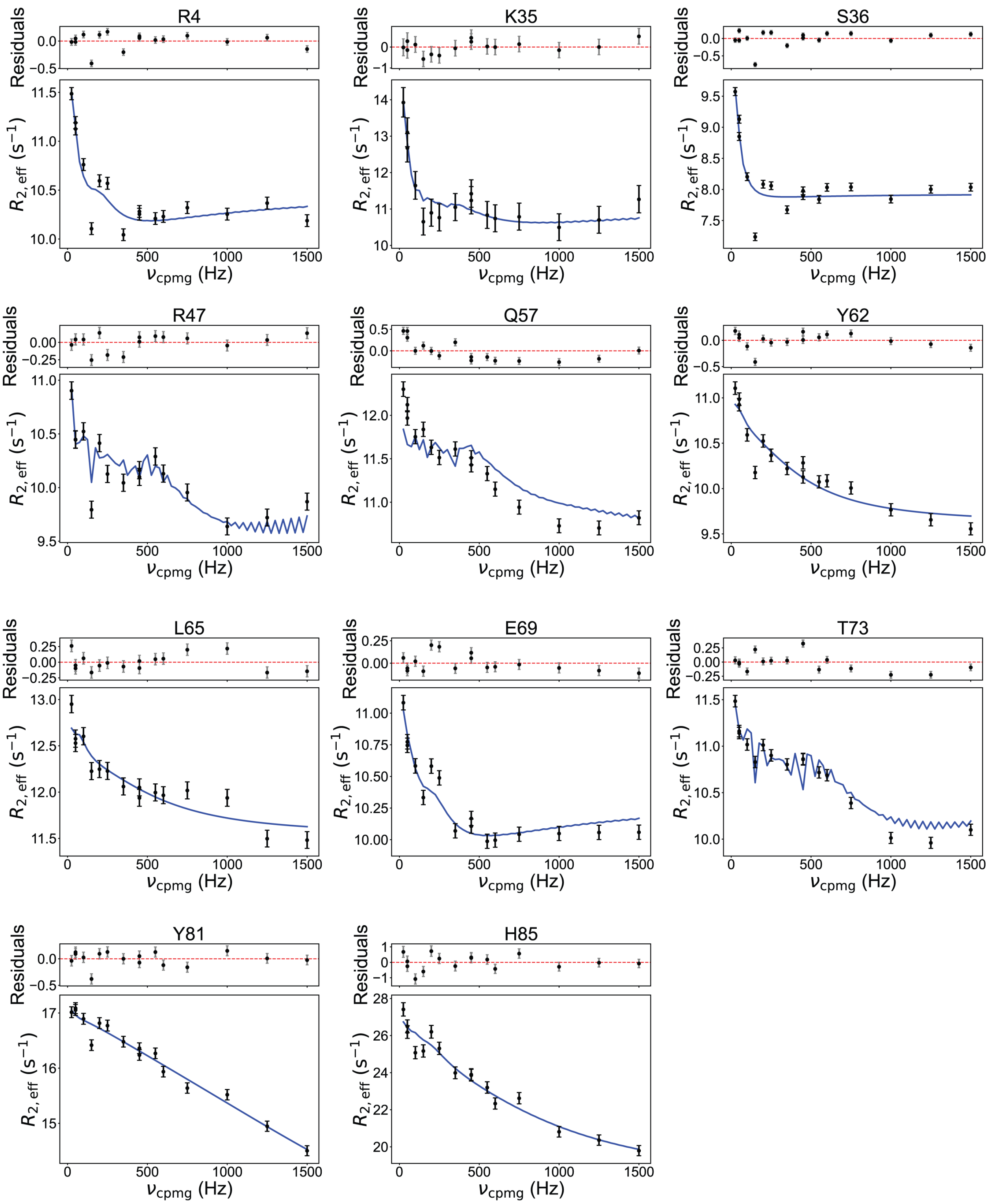

Fig. S18. ^{15}N CPMG profiles of residues in apo N-SH2^{T42A} fitted to 2-state exchange. (A) ^{15}N CPMG profiles of residues fitted to a 2-state exchange resulting in k_{ex} = 1260 ± 330 s^{-1}. **(B)** ^{15}N CPMG profiles of residues fitted to a 2-state exchange resulting in k_{ex} =>10000 s^{-1}. **(C)** Profiles of residues in apo N-SH2^{WT} that could not be fitted to a group (colored brown in **Fig. 5L**). These are highly dynamic exchange processes between two states, A and D. All datasets were collected with a 40 ms relaxation delay and at 25 °C.

A)

```
                 1                                                                66                                     103
9EH9/9MUQ     GSGMTSRRWFHPNITGVEAENLLLTRGVDGSFLARPSKSNPGDFTLSVRRNGAVTHIKIQNTGDYYDLYGGEKFATLAELVQYYMEHHGQLKEKNGDVIELKYPLNCAD
1AYD/9Y5X     --GSHMRRWFHPNITGVEAENLLLTRGVDGSFLARPSKSNPGDFTLSVRRNGAVTHIKIQNTGDYYDLYGGEKFATLAELVQYYMEHHGQLKEKNGDVIELKYPLN---
  /9Z7O
```

B)

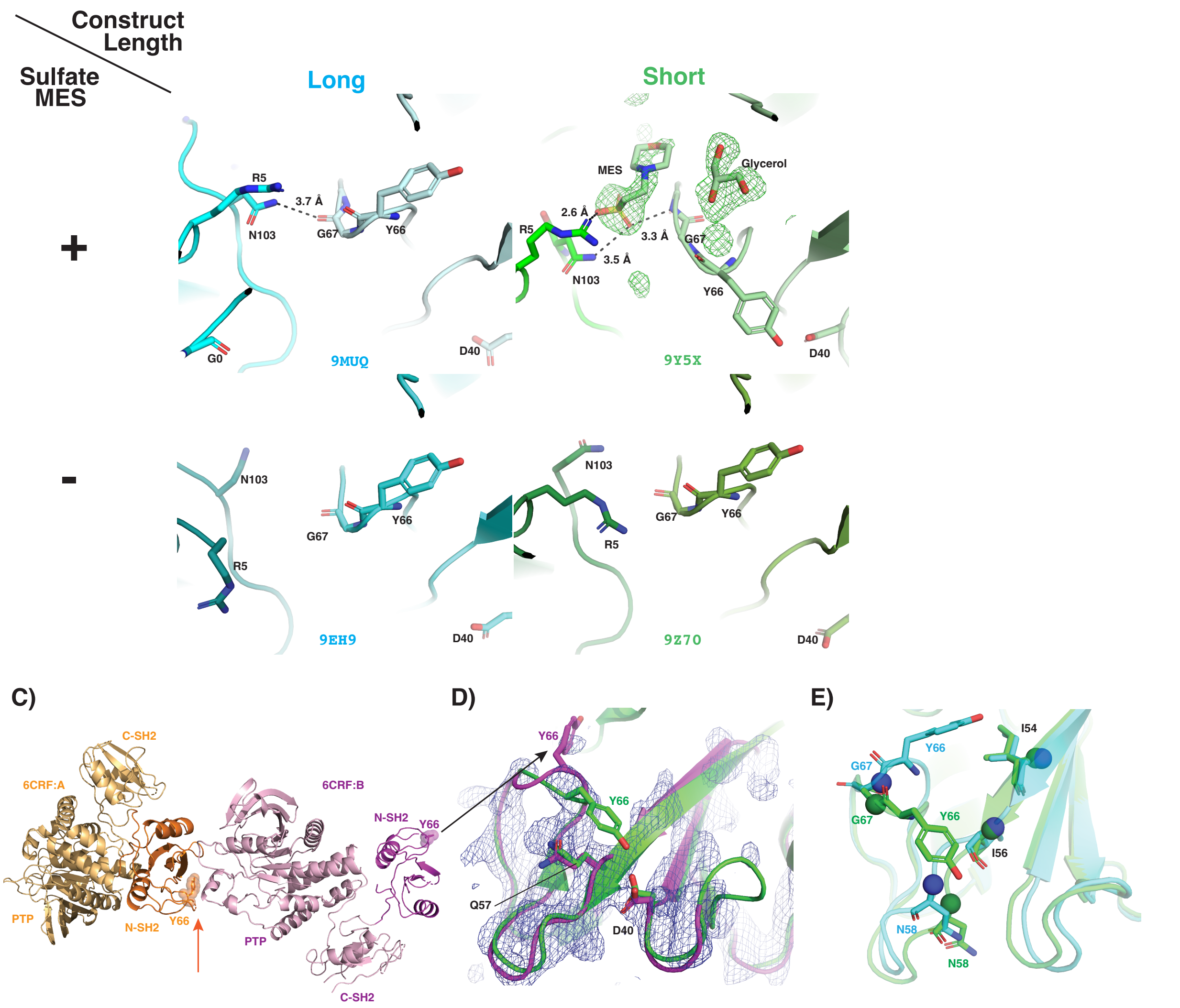

Fig. S19. The influence of crystallization condition and construct composition on the N-SH2^{WT} crystal structure and conformational states. In stark contrast to our unbound N-SH2^{WT} crystal structure (PDB 9EH9), inspection of the previously reported structure (PDB entry 1AYD (2)) revealed: i) the sidechain of Y66 adopts the same conformation as seen in the peptide-bound structures and ii) the presence of unmodelled additional electron density in the peptide binding site. To test whether the different crystallization conditions affected the conformation of the apo N-SH2^{WT}**,** we solved the structure using the reported 1AYD crystallization condition (PDB 9MUQ). Because the structures 9EH9 and 9MUQ turned out to be identical, the slight difference in the construct composition was investigated. **A)** Sequences of the construct used in this study (cyan, N-SH2^{WT}) and the 1AYD construct (green, N-SH2$^{WT\text{-}short}$). The remaining residues from the affinity tag after cleavage are shown in red. We reproduced the exact construct/crystallization conditions of 1AYD (deposited at 2.2 Å resolution) and solved the structure at 1.8 Å (deposited PDB 9Y5X). **(B)** Interactions between two adjacent molecules related by crystallographic symmetry involving the termini and EF-loop. Similarly to the 1AYD structure, additional positive electron density was observed between the asymmetric units of the 9Y5X crystal structure and in the peptide

binding pocket. Because the cryoprotectant solution contained 46 mM MES, 20% glycerol, and 833 mM ammonium sulfate, the electron density blobs were fitted with a MES and a glycerol molecule (green mesh, 3 σ contoured mFo-DFc-polder map (24)) with a calculated occupancy of 0.8. We note that the exact nature of the bound molecule is not certain given that MES possess a sulfonate moiety, similar to sulfate, and the electron density signal for the ring was weak. Zooming in on the crystal contacts revealed a weak hydrogen bond between the side chain of N103 and backbone of G67 in 9MUQ, but a salt bridge between the MES sulfonate moiety and R5, and hydrogen bonds of the sulfonate moiety with N103 and G67 in the 9Y5X lattice. Similarly, the N103 side chain in our structure replaces the sulfonate moiety found in 9Y5X. The extended N- and C-terminus in our construct placed the N103 in such a way that prevents the binding of the MES and enabled the apo conformation with the Y66 in the blocking conformation. To confirm our hypothesis, we obtained N-SH2$^{WT\text{-}short}$ crystals in a condition lacking sulfate/MES and solved the structure (PDB 9Z70). As expected, we found Y66 in the peptide blocking position proving that, in 1AYD entry, the choice of construct and presence of ligands from the crystallization solution shifted the equilibrium of N-SH2 domain towards a conformation similar to the peptide bound state. **(C)** To further investigate the conformations of Y66, we re-analyzed the electron density of full-length SHP2^{E76K} in the open state (PDB-REDO 6CRF). This structure is comprised of two molecules in the asymmetric unit (chain A, orange; chain B, magenta). The Y66 (orange spheres and arrow) of the N-SH2^{E76K} domain in chain A (dark orange) contacts the PTP domain of the neighboring molecule and adopts a similar conformation to the Y66 in 9Y5X. However, in chain B, the N-SH2^{E76K} (dark magenta) Y66 (dark magenta spheres) faced a solvent channel in the crystal. **(D)** In the deposited structure, the Y66 side chain for chain B is lacking due to weak electron density (6CRF)(25). PDB-REDO automatically placed the Y66 side chain in the blocking position for chain B (blue mesh, 1 σ contoured 2mFo-DFc map). Superposition with the 9Y5X structure (green) indicated that there is no electron density for Y66 in this alternative conformation. We note the absence of a clear electron density accounting for the Y66 sidechain in chain B of 6CRF, where Y66 is not constrained by crystal packing, points to a dynamic process that corroborates our X-ray crystallography and NMR results. **(E)** The observed movement of Y66 (9MUQ versus 9Y5X) would cause local ring current effects on nearby backbone amides (shown in spheres for group 1 residues from Fig. 5K). We hypothesize that this Y66 movement in free N-SH2^{WT} could be the structural basis for the measured ^{15}N-CPMG relaxation dispersion for group 1 residues (I54, I56, N58, G67; shown in Fig. 5K and Fig. S17A), consistent with µs–ms timescale exchange between blocking and alternative Y66 conformations.

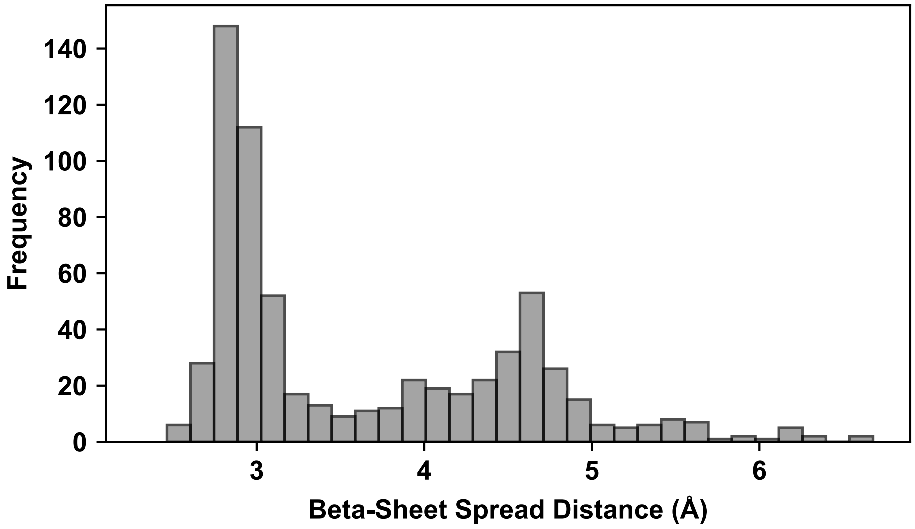

Fig. S20. Distribution of β-sheet distances across 660 human SH2 domain structures. Frequency of β-sheet spread distances, measured as the distance between residues corresponding to Phe41 and Ile56 in the SHP2 N-SH2. Two dominant conformations are observed: a zipped state (~3 Å) and unzipped conformations (with a maximum around 4.5 Å)

	N-SH2^{WT} Apo	N-SH2^{WT} p-Tyr	N-SH2^{WT} 1P-GAB1$_{N-term}$	N-SH2^{T42A} Apo	N-SH2^{T42A} 1P-GAB1$_{N-term}$	N-SH2^{WT} Apo (1AYD condition)	N-SH2$^{WT-Short}$ Apo (1AYD construct and condition)	N-SH2$^{WT-Short}$ Apo (1AYD construct no sulfate/MES)
**PDB Code**	**9EH9**	**9EHA**	**9EHD**	**9EIC**	**9EIK**	**9MUQ**	**9Y5X**	**9Z70**
**Resolution range (Å)**	42.75 - 1.83 (1.9 - 1.83)	36.91 - 1.71 (1.77 - 1.71)	37.43 - 1.59 (1.63 - 1.59)	35.89 - 1.58 (1.62 - 1.58)	37.82 - 1.25 (1.28 - 1.25)	41.34 - 1.8 (1.86 - 1.8)	31.64 - 1.795 (1.93 - 1.8)	47.14 – 1.73 (1.79-1.73)
**Space group**	$P4_32_12$	$P4_32_12$	$P4_32_12$	$P4_32_12$	$P4_32_12$	$P4_32_12$	$P4_32_12$	$P4_32_12$
**Unit cell (Å, °)**	60.5 60.5 78.3 90 90 90	58.6 58.6 81.3 90 90 90	61.5 61.5 73.5 90 90 90	56.7 56.7 80.3 90 90 90	62.1 62.1 74.4 90 90 90	58.47 58.47 80.86 90 90 90	63.27 63.27 76.28 90 90 90	57.8 57.8 81.4 90 90 90
**Unique reflections**	13292 (1420)	15891 (1406)	19583 (1369)	18599 (1392)	40860 (2847)	13578 (1321)	15043 (2930)	15009 (1333)
**Completeness (%)**	99.49 (97.73)	99.86 (99.08)	99.94 (99.85)	99.97 (99.71)	99.89 (99.58)	99.64 (98.56)	99.98 (99.97)	99.91 (99.55)
**Wilson B-factor**	49.86	34.5	34.77	28.62	21.33	42.79	37.54	30.75
**Reflections used in refinement**	13292 (1420)	15891 (1406)	19571 (1367)	18599 (1392)	40860 (2847)	13532 (1302)	15043 (2930)	15009 (1333)
**Reflections used for R-free**	1328 (142)	1590 (140)	1956 (137)	1860 (140)	2000 (139)	1353 (129)	763 (150)	1502 (133)
**R-work**	0.2381 (0.4567)	0.2027 (0.3566)	0.2002 (0.3094)	0.2081 (0.3357)	0.2009 (0.3118)	0.2176 (0.3790)	0.2119 (0.3687)	0.1945 (0.3646)
**R-free**	0.2579 (0.5428)	0.2442 (0.3252)	0.2392 (0.2992)	0.2250 (0.3214)	0.2286 (0.3595)	0.2654 (0.4302)	0.2508 (0.4077)	0.2325 (0.4336)
**Number of non-hydrogen atoms**	836	950	945	871	1004	886	973	914
**macromolecules**	811	866	904	815	893	840	895	867
**ligands**	4	5	0	10	0	11	28	0
**solvent**	21	79	41	46	111	35	50	47
**Protein residues**	101	105	110	102	111	104	104	104
**r.m.s.(bonds) (Å)**	0.01	0.007	0.008	0.007	0.009	0.011	0.012	0.008
**r.m.s. (angles) (°)**	1.03	0.95	1.1	1.04	1.14	1.02	1.40	1.05
**Ramachandran favored (%)**	98.99	98.04	99.03	97	96.15	99.02	92.16	98.04
**Ramachandran allowed (%)**	1.01	0.98	0.97	2	3.85	0.98	6.86	1.96
**Ramachandran outliers (%)**	0	0.98	0	1	0	0	0.98	0
**Rotamer outliers (%)**	3.49	1.11	0	0	0	0	2.13	1.09
**Clashscore**	5.6	9.42	2.26	5.56	2.29	6.59	10.48	3.49
**Average B-factor**	64.82	43.43	51.88	43.9	34.99	54.42	57.88	44.34
**macromolecules**	65.11	43.29	52.17	44.13	34.59	54.47	57.88	44.44
**ligands**	78.16	36.26		41.45		66.85	73.78	
**solvent**	51.19	45.43	45.43	40.35	38.22	49.42	48.94	42.41
**Ensemble refinement**								
**R-work**	0.1915	0.1768	0.1794	0.1864	0.1882			
**R-free**	0.2417	0.2175	0.2175	0.2143	0.2086			
**pTLS**	0.7	0.6	0.8	0.6	0.8			
**w-Xray**	5	10	10	2.5	2.5			
**tx**	1.2	1.4	0.8	0.8	1.3			
**Ensemble size**	89	200	34	200	50			

Table S1. **X-ray crystallography data collection and refinement statistics**. Values in parentheses are for the highest resolution shell.

Model	Experiment	$k_{ex,AB}$(s^{-1}); p_B (%)	$k_{ex,AC}$(s^{-1})	$k_{ex,BC}$(s^{-1})	X^2_{red}, BIC, AIC	residues
Unzipped A ↕ B Zipped	WT GAB1 (CEST) **Fig. 5B, S13A, S13B**	104+/-8.5;11+/-0.30 (brown)	-	-	0.9, -28, -137	W6, F7, G13, E15, K35, G39, D40, F41, T42, S44, V45, R46, R47, T52, H53, K55, I56, Q57 , T59, D61, L65, Y66, G67, E69, H84, L88, K89, D94, V95, I96
		45+/-5.0; 16+/- 0.60 (yellow)			0.9, -6.8, -47	L43, I54
Unzipped A ↔ B Zipped; A ↔ C Unzipped*	WT GAB1 (CPMG) **Fig. 5E, S15A-B**	(1). 104+/-8.5; 11+/-0.30 (fixed) (Brown)			1.6, 164, 96	T12, G13, G30, F41, T42, S44, V45, R46, R47, G60, D94
		104+/-8.5 (fixed)	(2). 2600+/-100; (Blue)	0	1.2, 67, 29	T52, I56, T59, Y66, L88, V95
		45+/-5.0	(2). 1830+/-400 (Blue)		0.14, -22, -25	I54
Unzipped A ↕ B Zipped ↕ C Unzipped*	WT GAB1 (CPMG)	104+/-8.5 (fixed)	0	(2). 1120+/-110	4.3, 182, 145	T52, I56, T59, Y66, L88, V95
Unzipped A ↔ B Zipped; A ↔ C Unzipped*; B ↔ C	WT GAB1 (CPMG)	104+/-8.5 (fixed)	(2). 2010+/-270	(2). 620 +/-60	1.2, 67, 27	T52, I56, T59, Y66, L88, V95
Unzipped A ↕ D Unzipped*	WT APO (CPMG) **Fig. 5K, Fig. S17A-B**	-	3550+/-320 (green)	-	4.2, 129, 107	I54, I56, N58, G67
			7000+/-670 (magenta)		5.5, 262, 218	Y62, L65, T73, Y81, M82, H84, L88
Unzipped A ↕ D Unzipped*	T42A APO (CPMG) **Fig. 5L, Fig. S18A-B**	-	1260+/- 330 (green)	-	3.6, 119, 97	I54, I56, N58, G67
			>10000 (magenta)			M82, H84, L88 Individually fitted

Table S2. Summary table of all fitted rate constants and populations from ^{15}N-CPMG and ^{15}N-CEST data, including alternate models with the corresponding statistical evaluation of x^2_{red}, BIC (Bayesian Information Criterion), AIC (Akaike Information Criterion) Data were obtained by fitting the CEST and CPMG data of the apo and 1P-GAB1$_{N\text{-}term}$-bound (denoted by GAB1 in the table) N-SH2 variants to either a 2-state or 3-state model. The numbers (1) and (2) are according to the groups in **the stated figures**. Colors noted in parentheses and experiment names match the color coding and structure labels in **Fig. 5**. Populations of states B and C derived from CPMG are underdetermined. All data were collected at 25 °C.

References

1. R. A. P. Padua *et al.*, Mechanism of activating mutations and allosteric drug inhibition of the phosphatase SHP2. *Nat Commun* **9**, 4507 (2018).
2. C. Lee *et al.*, Crystal Structures of peptide complexes of the amino-terminal SH2 domain of the Syp tyrosine phosphatase. *Stucture* **2** (1994).
3. N. J. Anthis, G. M. Clore, Sequence-specific determination of protein and peptide concentrations by absorbance at 205 nm. *Protein Sci* **22**, 851-858 (2013).
4. F. Delaglio *et al.*, NMRPipe: a multidimensional spectral processing system based on UNIX pipes. *J Biomol NMR* **6**, 277-293 (1995).
5. J. Ying, F. Delaglio, D. A. Torchia, A. Bax, Sparse multidimensional iterative lineshape-enhanced (SMILE) reconstruction of both non-uniformly sampled and conventional NMR data. *J Biomol NMR* **68**, 101-118 (2017).
6. D. F. Hansen, P. Vallurupalli, L. E. Kay, An improved 15N relaxation dispersion experiment for the measurement of millisecond time-scale dynamics in proteins. *J Phys Chem B* **112**, 5898-5904 (2008).

7. P. Vallurupalli, G. Bouvignies, L. E. Kay, Studying "invisible" excited protein states in slow exchange with a major state conformation. *J Am Chem Soc* **134**, 8148-8161 (2012).
8. M. Guenneugues, P. Berthault, H. Desvaux, A method for determining B1 field inhomogeneity. Are the biases assumed in heteronuclear relaxation experiments usually underestimated? *J Magn Reson* **136**, 118-126 (1999).
9. I. Manthey *et al.*, POKY software tools encapsulating assignment strategies for solution and solid-state protein NMR data. *J Struct Biol X* **6**, 100073 (2022).
10. M. Bieri, E. J. d'Auvergne, P. R. Gooley, relaxGUI: a new software for fast and simple NMR relaxation data analysis and calculation of ps-ns and mus motion of proteins. *J Biomol NMR* **50**, 147-155 (2011).
11. A. Ahlner, M. Carlsson, B. H. Jonsson, P. Lundstrom, PINT: a software for integration of peak volumes and extraction of relaxation rates. *J Biomol NMR* **56**, 191-202 (2013).
12. D. K. Schneider *et al.*, AMX - the highly automated macromolecular crystallography (17-ID-1) beamline at the NSLS-II. *J Synchrotron Radiat* **29**, 1480-1494 (2022).
13. W. Kabsch, Xds. *Acta Crystallogr D Biol Crystallogr* **66**, 125-132 (2010).
14. H. R. Powell, T. G. G. Battye, L. Kontogiannis, O. Johnson, A. G. W. Leslie, Integrating macromolecular X-ray diffraction data with the graphical user interface iMosflm. *Nat Protoc* **12**, 1310-1325 (2017).
15. P. R. Evans, G. N. Murshudov, How good are my data and what is the resolution? *Acta Crystallogr D Biol Crystallogr* **69**, 1204-1214 (2013).
16. A. J. McCoy *et al.*, Phaser crystallographic software. *J Appl Crystallogr* **40**, 658-674 (2007).
17. P. V. Afonine *et al.*, Towards automated crystallographic structure refinement with phenix.refine. *Acta Crystallogr D Biol Crystallogr* **68**, 352-367 (2012).
18. D. Liebschner *et al.*, Macromolecular structure determination using X-rays, neutrons and electrons: recent developments in Phenix. *Acta Crystallogr D Struct Biol* **75**, 861-877 (2019).
19. P. Emsley, B. Lohkamp, W. G. Scott, K. Cowtan, Features and development of Coot. *Acta Crystallogr D Biol Crystallogr* **66**, 486-501 (2010).
20. C. J. Williams *et al.*, MolProbity: More and better reference data for improved all-atom structure validation. *Protein Sci* **27**, 293-315 (2018).
21. B. T. Burnley, P. V. Afonine, P. D. Adams, P. Gros, Modelling dynamics in protein crystal structures by ensemble refinement. *Elife* **1**, e00311 (2012).
22. G. Kim *et al.*, Easy and accurate protein structure prediction using ColabFold. *Nat Protoc* **20**, 620-642 (2025).
23. D. Bajusz, G. Pandy-Szekeres, A. Takacs, E. D. de Araujo, G. M. Keseru, SH2db, an information system for the SH2 domain. *Nucleic Acids Res* **51**, W542-W552 (2023).
24. D. Liebschner *et al.*, Polder maps: improving OMIT maps by excluding bulk solvent. *Acta Crystallogr D Struct Biol* **73**, 148-157 (2017).
25. J. R. LaRochelle *et al.*, Structural reorganization of SHP2 by oncogenic mutations and implications for oncoprotein resistance to allosteric inhibition. *Nature Communications* **9** (2018).
